# Transcriptional Profiling of *Saccharomyces cerevisiae* Reveals the Impact of Variation of a Single Transcription Factor on Differential Gene Expression in 4NQO, Fermentable, and Nonfermentable Carbon Sources

**DOI:** 10.1534/g3.117.300138

**Published:** 2017-12-05

**Authors:** Xiaoqing Rong-Mullins, Michael C. Ayers, Mahmoud Summers, Jennifer E. G. Gallagher

**Affiliations:** Department of Biology, West Virginia University, Morgantown, West Virginia 26506

**Keywords:** Yrr1, 4NQO, RNA-Seq, ChIP-Seq, genetic variation, respiration, fermentation

## Abstract

Cellular metabolism can change the potency of a chemical’s tumorigenicity. 4-nitroquinoline-1-oxide (4NQO) is a tumorigenic drug widely used on animal models for cancer research. Polymorphisms of the transcription factor Yrr1 confer different levels of resistance to 4NQO in *Saccharomyces cerevisiae*. To study how different Yrr1 alleles regulate gene expression leading to resistance, transcriptomes of three isogenic *S*. *cerevisiae* strains carrying different Yrr1 alleles were profiled via RNA sequencing (RNA-Seq) and chromatin immunoprecipitation coupled with sequencing (ChIP-Seq) in the presence and absence of 4NQO. In response to 4NQO, all alleles of Yrr1 drove the expression of *SNQ2* (a multidrug transporter), which was highest in the presence of 4NQO resistance-conferring alleles, and overexpression of *SNQ2* alone was sufficient to overcome 4NQO-sensitive growth. Using shape metrics to refine the ChIP-Seq peaks, Yrr1 strongly associated with three loci including *SNQ2*. In addition to a known Yrr1 target *SNG1*, Yrr1 also bound upstream of *RPL35B*; however, overexpression of these genes did not confer 4NQO resistance. RNA-Seq data also implicated nucleotide synthesis pathways including the *de novo* purine pathway, and the ribonuclease reductase pathways were downregulated in response to 4NQO. Conversion of a 4NQO-sensitive allele to a 4NQO-resistant allele by a single point mutation mimicked the 4NQO-resistant allele in phenotype, and while the 4NQO resistant allele increased the expression of the *ADE* genes in the *de novo* purine biosynthetic pathway, the mutant Yrr1 increased expression of *ADE* genes even in the absence of 4NQO. These same *ADE* genes were only increased in the wild-type alleles in the presence of 4NQO, indicating that the point mutation activated Yrr1 to upregulate a pathway normally only activated in response to stress. The various Yrr1 alleles also influenced growth on different carbon sources by altering the function of the mitochondria. Hence, the complement to 4NQO resistance was poor growth on nonfermentable carbon sources, which in turn varied depending on the allele of Yrr1 expressed in the isogenic yeast. The oxidation state of the yeast affected the 4NQO toxicity by altering the reactive oxygen species (ROS) generated by cellular metabolism. The integration of RNA-Seq and ChIP-Seq elucidated how Yrr1 regulates global gene transcription in response to 4NQO and how various Yrr1 alleles confer differential resistance to 4NQO. This study provides guidance for further investigation into how Yrr1 regulates cellular responses to 4NQO, as well as transcriptomic resources for further analysis of transcription factor variation on carbon source utilization.

*Saccharomyces cerevisiae*, baker’s yeast, is a model organism that has been extensively studied to decipher the association between genotypes and phenotypes. In addition to the widely used laboratory strain S288c (Engel *et al.* 2014), the genomes of other yeast strains have been sequenced, which provides valuable resources to investigate how the genetic differences among strains contribute to phenotypic differences. The YJM789 yeast strain was derived from a clinical isolate from an AIDS patient with *S*. *cerevisiae* pneumonia, and its draft genome is available (Wei *et al.* 2007). Genome association comparison of YJM789 to S96, a strain derived from S288c, elucidated the genetic basis of strain-dependent responses to a toxic chemical, 4NQO (Gallagher *et al.* 2014).

4NQO is a quinoline-derived carcinogenic drug used for cancer research on animal models because it induces squamous cell carcinoma in oral cavities of mice (Hawkins *et al.* 1994). YJM789 shows higher resistance to 4NQO treatment than S96 does in Yeast Peptone Dextrose (YPD) medium (Gallagher *et al.* 2014). Mapping of quantitative trait loci shows that the difference in resistance to 4NQO between the two strains was correlated with variation in the *YRR1* gene, which encodes a zinc finger transcription factor. The Yrr1 protein from YJM789, Yrr1^Y^, possesses a threonine at the position of 775 (T775), while the Yrr1 protein from S96, Yrr1^S^, possesses an isoleucine at this position (I775). Threonine can be phosphorylated at its hydroxyl functional group, while isoleucine does not possess a hydroxyl group and thus cannot be phosphorylated. However, phosphorylation could not be confirmed, likely due to the technical limitations of detection using mass spectrometry. The peptide containing the potential phosphorylation is 4578 Da and the average weight of the peptides is 1700 Da, and therefore would be considered too large for detection (Rong-Mullins *et al.* 2017b). Combined with low abundance of transcription factors and the length of the peptide, this precludes the validation of this phosphorylation. Instead, a mutant protein was constructed, Yrr1^IE^, that mimics the strong negative charge of phosphorylation when I775 of the S96 allele is changed into a glutamate (I775E). The level of 4NQO resistance conferred by Yrr1^IE^ is comparable to that conferred by Yrr1^Y^ and is higher than that conferred by Yrr1^S^. Conversely, a single point mutation E673G in Yrr1^Y^ can convert the 4NQO-resistant allele to a 4NQO-sensitive allele. However, combining E673G with T775E retains 4NQO resistance conferred by Yrr1^Y^. This suggests that the potential phosphorylation at T775 of Yrr1^Y^ may play an important role in regulating cellular responses to 4NQO.

YJM789 was isolated from a patient with multiple viral infections being treated for pneumonia with ciprofloxacin, an antibacterial fluoroquinolone (Tawfik *et al.* 1989). *S. cerevisiae* is associated with the normal human microbiome and is typically not considered to be pathogenic. However, YJM789’s original heterozygous diploid parent adapted to unique conditions, including a suppressed immune system and a lack of bacterial competition. It is unknown if ciprofloxacin treatment directly contributed to the 4NQO tolerance seen in this strain, if the tolerance preexisted, or if the tolerance came about by adapting to another condition. Several mutations within Yrr1 that changed cellular growth in response to 4NQO also changed cellular growth when cells were forced to respire (Gallagher *et al.* 2014).

The mechanisms of transcription regulation by Yrr1 have been investigated in earlier studies regarding drug resistance. Yrr1 was shown previously to autoregulate by binding to its own promoter region (Zhang *et al.* 2001). In addition, Yrr1 is also known to induce higher expression of *SNQ2*, a gene encoding a multidrug transporter, in response to 4NQO (Cui *et al.* 1998; Le Crom *et al.* 2002). However, genome-wide changes of transcription and Yrr1-binding patterns have not been reported to provide a comprehensive view. In this study, we constructed three isogenic strains in the *yrr1*Δ S96 background carrying three different Yrr1 alleles: Yrr1^S^, Yrr1^Y^, and Yrr1^IE^. We then conducted deep RNA-Seq as well as ChIP-Seq in conditions where Yrr1 regulates the stress response in the presence of 4NQO and glycerol. Here, we discovered that overexpression of *SNQ2* bypassed the need for Yrr1 for 4NQO sensitivity conferred by expression of the Yrr1^S^ allele. While the expression of many genes changed when cells with different alleles of Yrr1 were grown in glycerol, differences in the purine salvage pathway and other antioxidant pathways may quench free radicals generated as yeast shift toward respiration. In the model presented here, 4NQO was actively reduced in the presence of functioning mitochondria to produce free radicals and 4HAQO. 4NQO in the form of 4HAQO interacts directly with DNA to induce DNA oxidation. Our data indicate that genetic variation in a single transcription factor altered the process of oxidative phosphorylation within the cell, which accounted for the aforementioned conversion of 4NQO into a toxic mixture of free radicals and 4HAQO. This study will not only broaden our knowledge of the yeast metabolic response to tumorigenic drugs, but also inform research on the drug resistance of cancer cells for other model organisms. Furthermore, the data presented here provide a resource for the further exploration of the effect of genetic variation in a transcription factor on the utilization of carbon sources.

## Materials and Methods

### S. cerevisiae strains and plasmids

The strains and plasmids used in this study were described in Gallagher *et al.* (2014) and grown in YPD (1% yeast extract, 2% peptone, and 2% dextrose). Plasmids encoding alleles of *YRR1* were under the control of endogenous promoters and terminators in pGS35, and were maintained by the addition of G418. The entire coding region of *YRR1* was replaced by the hygromycin-resistance gene in S96 (*MATa*, *lys5*) and in FY3, an isogenic yeast strain (*MATa*, *ura3*) (Winston *et al.* 1995; Goldstein and McCusker 1999; Gallagher *et al.* 2014). Petite strains were generated by treating parent stains (FY3 *yrr1*::*URA3*, YJM789, YJM789 *yrr1*::*HYG^R^*, S96, and S96 *yrr1*::*HYG^R^*) with 1 μg/ml of ethidium bromide for 6 hr in a liquid culture and plating on YPD. After 2 d, the colonies were replica-plated onto YP with 3% glycerol as the sole carbon source, and colonies that failed to grow were tested for loss of the mitochondrial-encoded *COX2* gene by PCR amplification. For serial dilution growth assays, saturated cultures were grown in YPD, serially diluted 10-fold, and spotted onto the indicated media (Rong-Mullins *et al.* 2017a). Because the S96 strain is an isogenic strain to S288c, the sequences and annotations of S288c genome release R64-1-1 (Engel *et al.* 2014) were used for RNA-Seq and ChIP-Seq analyses. *SNQ2* and *PDR5* overexpression plasmids were previously published (Tsujimoto *et al.* 2015). Overexpression plasmids were based on yEP24 with the *URA3* selectable marker and were transformed into FY3 *yrr1*Δ with pGS35. Plasmids were maintained on Yeast Minimal (YM) media with no amino acids and monosodium glutamate as the nitrogen source, so that 500 μg/ml G418 would select for pGS35 (Kan^R^ marker). Overexpression plasmids of all other strains were from the MORF collection (Gelperin *et al.* 2005). Plasmids were maintained in FY3 *yrr1*Δ by selecting for the *URA3* auxotrophy. Expression of the MORF collection is driven by the *GAL* promoter. Yeast were grown in YP with 2% galactose and 0.1% dextrose to prevent toxicity from overexpression. In YP glycerol (abbreviated as Glyc), 2% galactose and 3% ethanol were also added to the solid media.

### RNA-Seq experiment and data analysis

Each of the three untagged isogenic strains with different alleles—Yrr1^S^, Yrr1^Y^ and Yrr1^IE^—were grown as duplicated cultures in liquid YPD medium with G418 to midlog phase. Each culture was diluted to early log phase and divided into two subcultures. One subculture was incubated in liquid YPD medium with 0.25 µg/ml 4NQO, and the other subculture was incubated without 4NQO or washed and diluted into 3% glycerol. Subcultures for each replicate were further grown for 2.5 or 10 hr, respectively. Total RNA was extracted from each subculture (sample) and multiplexed. Unstranded paired-end cDNA libraries were prepared using an Illumina TruSeq RNA Sample Prep Kit v2. The libraries were sequenced on Illumina MiSeq to generate 1.30–1.99 million 76 bp read pairs for each sample. All the software parameters used in RNA-Seq analysis were set to default unless specified otherwise.

Tophat v2.0.12 (Trapnell *et al.* 2009; Kim *et al.* 2013) was used to map the RNA-Seq reads to the S288c genome and to generate .bam files for the mapped reads. The reads were first mapped to ribosomal RNA (rRNA) and transfer RNA genes (tRNA); 1.05–1.94 million read pairs per sample were not matched to those RNA genes, and 91.2–95.5% of those unmatched reads were then matched to the whole gene pool of S288c. The reads matched to rRNA and tRNA were excluded because these RNAs tend to be extracted at high and/or variable abundances across replicates, thus may bias the calculation of FPKM (fragments per kilobase gene per million mapped fragments). The R-package Rsubread v1.18.0 (Liao *et al.* 2013) was used to generate count tables based on the annotations of S288c genome release R64-1-1.

DESeq2 v1.8.1 (Anders *et al.* 2010) was used for differential expression calculation. A *q*-value (*p*-value adjusted for multi-testing) < 0.05 was considered significant for differential expression. In addition, noncoding RNA such as small nucleolar RNA genes were quantified but not considered in analyses of differential expression or statistical relevance (to ChIP-Seq data). This is because many of those genes showed low and/or variable FPKM values across replicates; hence, their quantification results may not accurately reflect their actual cellular abundance. Log_2_(fold change) of FPKM was calculated between all the pair-wise contrasts of conditions with a difference in only one of the following two factors: allele and growth medium [(YPD, YPD + 4NQO (abbreviated as 4NQO), and Glyc]. It was also calculated between the trial of Yrr1^IE^ in YPD and the trial of Yrr1^Y^ in 4NQO or Glyc to test whether the phosphomimic mutation of Yrr1^IE^ in an absence of 4NQO (or when cells are grown in Glyc) results in transcriptional states similar to that of Yrr1^Y^ in the presence of 4NQO.

### Gene ontology (GO) analysis

Significantly upregulated and downregulated genes were extracted from the Deseq2 output using MATLAB R2016a. Their significance was determined using a *p*-value cutoff of 0.05. Subsequently, the GO terms of the significant genes were determined using the Yeastmine database (Engel *et al.* 2014). In order to mitigate the issue of multiple comparisons and reduce the familywise error rate, the Holm–Bonferroni test correction (Hochberg 1988) was utilized with a *p*-value threshold of 0.05.

### ChIP-Seq data analysis

The ChIP-Seq data using the three allelic proteins of Yrr1 was previously generated (Gallagher *et al.* 2014). The three Myc-tagged isogenic strains with different alleles—Yrr1^S^, Yrr1^Y^ and Yrr1^IE^—were subjected to the same treatments as those in the RNA-Seq experiment. Three replicate cultures were prepared for each allele and treatment combination; immunoprecipitated DNA was sequenced for all three cultures in each combination, and input genomic DNA was sequenced for one culture per combination. The ChIP-enriched and input DNA samples were sequenced as multiplexed 101 bp paired-end libraries on an Illumina HiSequation 2000. From this, 2.08–4.21 million read pairs were generated per sample and mapped to the S288c genome using Bowtie v2.2.3 (Langmead and Salzberg 2012) with preset “–sensitive,” resulting in alignment rates of 96.1–99.3%. Reanalysis of the data is described below. ChIP-enriched regions (peaks) were identified using CisGenome v2.0 (Ji *et al.* 2008) and MACS2 v2.1.0 (Zhang *et al.* 2008), and their outputs were integrated for downstream analyses (see details in the supplemental material). Prediction of DNA motifs at potential Yrr1-binding sites was performed using the Gibbs Motif Sampler in CisGenome with some manual adjustment. Sequence logos of predicted motifs were generated using WebLogo v3.4 (Schneider and Stephens 1990; Crooks *et al.* 2004). The height of each nucleotide letter represented the posterior mean relative entropy, and the error bars represented Bayesian 95% C.I.s. Pearson’s correlation (*r*-value) test was performed on ChIP peak metrics of three consolidated peak regions and expression values of their downstream genes using the python scipy package.

### Microscopy

Cells were grown to midlog in YM and stained live using Mitotracker Green and Rhodamine B hexyl ester (Y7530; Life Technologies) per the manufacturer’s instructions. Cells were imaged for differential interference contrast and fluorescence microscopy using an Eclipse 600-FN Nikon microscope with an Apochromat 100×/1.40 NA oil immersion objective, and a cooled charge-coupled device camera (ORCA-2; Hamamatsu Photonics). Images were processed with MetaMorph v7.0 software (Molecular Devices) and further processed using Image J.

### Data availability

The *S. cerevisiae* strains used in this study are available upon request. Supplemental Material, File S1 describes the process of determining confidence in ChIP-Seq peaks based on peak shape metrics, as well as all the supporting figure legends and tables. The RNA-Seq and ChIP-Seq data used in this study are available at NCBI GEO with accession numbers GSE74642 and GSE74700, respectively. The links for the two data sets are: https://www.ncbi.nlm.nih.gov/geo/query/acc.cgi?acc= GSE74642 and https://www.ncbi.nlm.nih.gov/geo/query/acc.cgi?acc=GSE74700.

## Results and Discussion

### A global view of RNA-Seq transcriptomic profiles for different YRR1 alleles in the presence or absence of 4NQO

To determine the global transcriptional change due to the presence of different alleles of Yrr1, a transcription factor, the alleles were expressed in the same genetic background and treated with 4NQO. A total of 6717 open reading frames (ORFs) were quantified for FPKM in RNA-Seq. Ten comparisons of conditions were performed to identify differentially expressed ORFs (Table 1, Table S1, and Table S2). The within-allele comparisons between treatments of YPD (in the absence of 4NQO) and 4NQO for each Yrr1 allele were among those showing the largest numbers of significantly differentially expressed loci, with 510–909 loci significantly up- or downregulated (Table 1 and Table S2). Stress from 4NQO treatment induced the most dramatic transcriptomic changes. Interestingly, the comparison between Yrr1^S^ and Yrr1^Y^ in the presence of 4NQO showed the smallest number of significantly differentially expressed loci, with only six loci significantly up regulated and six downregulated (Table 1 and Table S2). In contrast, there were larger numbers of significantly differentially expressed loci when Yrr1^S^ or Yrr1^Y^ was compared to the phosphomimic allele Yrr1^IE^ in 4NQO, with 108–143 loci significantly up- or downregulated (Table 1). This suggests that the transcriptomic profile of Yrr1^IE^ was divergent from those of Yrr1^S^ or Yrr1^Y^ in 4NQO. Cells carrying Yrr1^IE^ had similar 4NQO resistance to Yrr1^Y^ cells, both of which were higher than that of Yrr1^S^ cells (Gallagher *et al.* 2014). Because Yrr1^IE^ was predicted to mimic the charge of phosphorylation at T775 of Yrr1^Y^ in response to 4NQO, the transcriptomic profile of Yrr1^IE^ in YPD was compared to that of Yrr1^Y^ in 4NQO. There were substantial numbers of loci significantly upregulated (671) and downregulated (602) in this comparison. This suggests that despite I775E mimicking the 4NQO resistance of Yrr1^Y^, Yrr1^IE^ was not simply an activated form of Yrr1^Y^. Despite the I775E mutation having the same phenotype as Yrr1^Y^ on 4NQO and our prediction that it would behave as an irremovable phosphorylation, the transcriptomic profile of Yrr1^IE^ in YPD was not similar to that of Yrr1^Y^ in 4NQO. Therefore, the phosphomimic Yrr1^IE^ allele of Yrr1^Y^ in 4NQO could not recapitulate all cellular effects of the YJM789 allele for several possible reasons, such as variation in other proteins, differences in phosphorylation kinetics, or incomplete phosphorylation of the cellular pool of the wild-type allele.

**Table 1:** The numbers of loci showing significant (*q*-value < 0.05) differential expression in RNA-Seq are shown as the total of each functional category and for each comparison of conditions (combination of *YRR1* allele and growth medium)

Functional Category*^a^*	Total Per Category	Expression Change	S96, 4NQO/YPD	YJM789, 4NQO/YPD	S96-I775E, 4NQO/YPD	YJM789/S96, 4NQO	S96-I775E/S96, 4NQO	S96-I775E/YJM789, 4NQO	YJM789/S96, YPD	S96-I775E/S96, YPD	S96-I775E/YJM789, YPD	S96-I775E YPD/YJM789 4NQO
All loci tested	6717	Up	750	510	609	6	116	108	55	390	385	671
		Down	909	605	760	6	143	118	10	252	354	602
DNA damage	328	Up	31	24	24	0	2	3	0	9	7	16
		Down	27	23	19	0	2	4	0	5	12	27
Oxidative stress	106	Up	44	35	32	0	2	2	4	24	18	7
		Down	12	7	11	0	11	10	0	5	7	33
Protein folding	116	Up	2	0	28	24	1	8	2	12	20	26
		Down	2	0	19	4	0	15	3	11	4	13
Purine biosynthesis and RNR genes	64	Up	14	8	7	0	3	1	0	1	1	13
		Down	24	20	15	1	6	4	0	16	17	12
Pyrimidine biosynthesis	19	Up	2	1	2	0	1	0	0	1	2	5
		Down	8	5	6	0	0	0	0	2	0	2
Nucleotide salvage	18	Up	1	1	1	0	0	0	0	1	2	4
		Down	9	3	6	0	0	1	0	2	1	1

The condition before “/” is the numerator and the one after is the denominator for differential expression. 4NQO, 4-nitroquinoline-1-oxide; YPD, Yeast Peptone Dextrose; RNR, ribonucleotide-diphosphate reductase.

a The genes in specific functional categories were found through Gene Ontology (GO) term search within *S. cerevisiae* on AmiGO2 (http://amigo2.geneontology.org/amigo): DNA damage: GO:0006974 representing “cellular response to DNA damage stimulus”; Oxidative stress: GO:0006979 representing “response to oxidative stress”; Protein folding: GO:0006457 representing “protein folding”; Purine biosynthesis and RNR genes: GO:0006164 representing “purine nucleotide biosynthetic process,” as well as *RNR1*, *RNR2*, *RNR3*, and *RNR4* genes; Pyrimidine biosynthesis: GO:0006221 representing “pyrimidine nucleotide biosynthetic process”; and Nucleotide salvage: union of GO:0043173 representing “nucleotide salvage,” GO:0043101 representing “purine-containing compound salvage,” and GO:0008655 representing “pyrimidine-containing compound salvage.”

### Genes involved in DNA damage and oxidative stress responses showcase remarkable patterns of differential expression in response to 4NQO

4NQO and its cellular metabolites are known to cause DNA damage in eukaryotes (Arima *et al.* 2006; Minca and Kowalski 2011). Among the 328 genes involved in the cellular response to a DNA damage stimulus, 19–31 of them were significantly up- or downregulated in response to 4NQO depending on the Yrr1 allele (Table 1). The upregulated genes encode endonucleases, helicases, and proteins involved in DNA mismatch repair. The downregulated genes encode proteins including histones, components of chromatin remodeling complexes, DNA replication proteins, RNA polymerase II subunits, and transcription initiation proteins. Changes in the gene expression of these pathways could compensate for 4NQO-induced damage to DNA.

Metabolic reduction of 4NQO in mammalian cells is known to generate ROS (Varnes and Biaglow 1979; Arima *et al.* 2006), and therefore imposes oxidative stress on cells. Among the 106 genes involved in the response to oxidative stress, 32–44 of them were significantly upregulated in response to 4NQO, depending on which Yrr1 allele was expressed (Table 1). These genes encode antioxidant proteins such as catalases, superoxide dismutases, peroxidases, peroxiredoxins, and thioredoxins. The higher abundance of these transcripts likely led to more effective reduction of ROS and thus alleviation of 4NQO-induced oxidative stress.

### Several differentially expressed genes may contribute to differential 4NQO resistance conferred by different Yrr1 alleles

**Figure 1:**
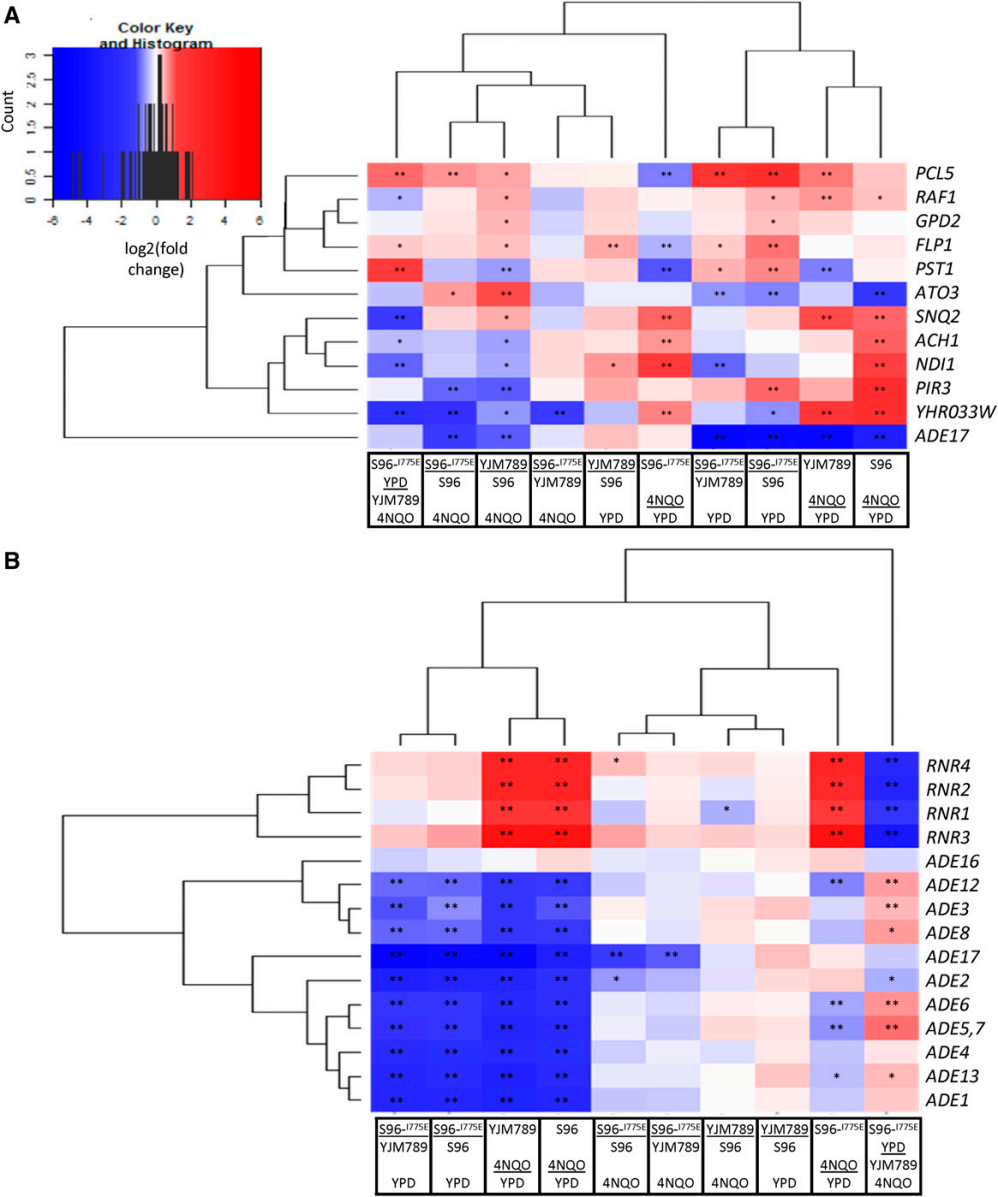
Heatmap of genes showing significant differential expression between alleles of Yrr1 in the presence of 4NQO. Log_2_(fold change of FPKM) values calculated by DESeq2 are represented in blue and red. Significant differential expression instances are highlighted based on *q*-values (*p*-values adjusted for multi-testing), where “*” represents a *q*-value between 0.005 and 0.05 and “**” represents < 0.005. (A) Levels of RNA that showed the strongest change in expression between Yrr1^S96^ and Yrr1^YJM789^. (B) Levels of RNA involved in nucleotide metabolism that changed between alleles of Yrr1. 4NQO, 4-nitroquinoline-1-oxide; FPKM, fragments per kilobase gene per million mapped fragments; YPD, Yeast Peptone Dextrose medium.

A tractable set of 12 genes were significantly differentially expressed between the S96 and YJM789 alleles of Yrr1 in the presence of 4NQO. It was hypothesized that these genes may be implicated in the differential resistance of the two alleles, prompting further examination (Figure 1). It is possible that subtle transcriptional differences of other genes between Yrr1^S^ and Yrr1^Y^ in the presence of 4NQO, despite being deemed not significant by our study, also contribute to the differential 4NQO resistance conferred by the two alleles.

When considering the 12 genes with significant expression differences between S96 and YJM789 alleles of Yrr1 under 4NQO treatment, we examined their known functions and potential relationships to 4NQO or other chemical toxins. Several had roles consistent with the putative interplay of resistance and carbon metabolism. Snq2 is an ABC plasma membrane transporter that is required for resistance to many chemicals, including 4NQO (Servos *et al.* 1993) and ROS (Ververidis *et al.* 2001). It is also known to be regulated by Yrr1 (Cui *et al.* 1998; Le Crom *et al.* 2002), so its differential expression indicates the detection capability of the study. *NDI1* encodes an NADH:ubiquinone oxidoreductase and is a component of the electron transport chain (ETC) in aerobic respiration. *ACH1* encodes a protein with CoA transferase and acetyl-CoA-hydrolase activities that appears in both the cytosol and mitochondria (Lee *et al.* 1990; Fleck and Brock 2009). Expression of *NDI1* and *ACH1* was significantly lower for Yrr1^Y^ than for Yrr1^S^ in the presence of 4NQO, because *NDI1* and *ACH1* were significantly upregulated for Yrr1^S^ but not for Yrr1^Y^ in response to 4NQO (Figure 1). Overexpression of *NDI1* is known to increase the accumulation of ROS (Li *et al.* 2006). Given that metabolic reduction of 4NQO generates ROS, such an expression pattern of *NDI1* may result in less accumulation of ROS, or change oxidative phosphorylation in the cell and decrease the reduction of 4NQO, thus conferring higher 4NQO resistance in cells carrying Yrr1^Y^ (Gallagher *et al.* 2014). In contrast to *NDI1*, deletion of *ACH1* was reported to decrease resistance to oxidative stress caused by 3 mM hydrogen peroxide (Brown *et al.* 2006), suggesting that higher levels of *ACH1* may lead to higher resistance to oxidative stress. Interestingly, our data suggests an association of higher levels of *ACH1* with lower resistance to oxidative stress: cells carrying Yrr1^S^, where *ACH1* expression is higher than in cells carrying Yrr1^Y^, are less resistant to 4NQO.

### Nucleotide biosynthetic genes

The genes involved in purine nucleotide biosynthetic processes showcased interesting patterns of differential expression, both in response to 4NQO and among Yrr1 alleles (Figure 1B and Table 1). Two subgroups, *ADE* genes and ribonucleotide-diphosphate reductase (*RNR*) genes, displayed relatively consistent patterns within each subgroup but distinct patterns from each other (Figure 1B and Table 1). In addition, null mutations of some genes from the two subgroups have opposite effects on resistance to oxidative stress, as described later.

Ade proteins catalyze the *de novo* purine biosynthetic steps from 5-phospho-ribosyl-pyrophosphate to inosine monophosphate (IMP) (Ade1, Ade2, Ade3, Ade4, Ade5,7, Ade6, Ade8, Ade16, and Ade17) and from IMP to adenosine monophosphate (Ade12 and Ade13). All the *ADE* genes except *ADE16* were significantly downregulated in response to 4NQO for Yrr1^S^ and Yrr1^Y^, and significantly lower for Yrr1^IE^ than Yrr1^S^ and Yrr1^Y^ in YPD (Figure 1B). Both Ade16 and Ade17 have 5-aminoimidazole-4-carboxamide ribonucleotide transformylase and IMP cyclohydrolase activities (Tibbetts and Appling 1997, 2000). *ADE16* is a paralog of *ADE17* with overlapping roles; the expression change of *ADE17* alone may be sufficient for achieving the necessary changes in metabolic activities catalyzed by both *ADE16* and *ADE17*. Like the protein-folding genes discussed above, the expression pattern of *ADE* genes for Yrr1^IE^ in YPD mimicked those of Yrr1^S^ and Yrr1^Y^ in 4NQO. Single null mutations of *ADE1*, *ADE3*, *ADE4*, *ADE5*, *ADE7*, and *ADE8* were shown to increase resistance to oxidative stress caused by 3 mM hydrogen peroxide (Brown *et al.* 2006). The downregulation of *ADE* genes in response to 4NQO, as shown in our data, may increase resistance to oxidative stress caused by 4NQO.

The RNR complex catalyzes the formation of dNDP from NDP, a rate-limiting step in dNTP synthesis. All four *RNR* genes were significantly upregulated in response to 4NQO for all three Yrr1 alleles (Figure 1B). This is consistent with a previous finding that the mRNA levels of *RNR1* and *RNR3* increase upon treatment with 4NQO, which likely facilitates DNA repair during replication (Elledge and Davis 1990). Deletion of *RNR1* was shown to decrease resistance to oxidative stress caused by 2 mM hydrogen peroxide, 0.5 mM paraquat, and 100% oxygen atmosphere (Outten and Culotta 2005). It is possible that upregulation of *RNR* genes copes with the oxidative stress caused by 4NQO.

### Identification of ChIP-Seq peaks with high confidence of being functional binding sites of Yrr1

In order to investigate binding of Yrr1 to DNA as a transcription factor in association with its impact on gene expression, the published ChIP-Seq data of Yrr1 (Gallagher *et al.* 2014) were reexamined together with the RNA-Seq data in this study (Table S3). In *S. cerevisiae*, transcription factors bind to specific recognition sequences upstream of genes, known as upstream activation or repression sequences (Phillips and Hoopes 2008; Hahn and Young 2011). No transcription factor is known to have recognition sequences within gene bodies. However, 1083 out of 1136 narrow peak regions (defined in the *Materials and Methods* and supplemental material) of Yrr1 ChIP enrichment identified by CisGenome and MACS2 overlap with annotated genes in the published S288c genome. False-positive ChIP peaks have been previously identified within highly expressed genes, *i.e.*, “hyper-ChIPable” regions (Teytelman *et al.* 2013). Therefore, to examine whether hyper-ChIPability exists in our Yrr1 ChIP-Seq data, expression levels of loci overlapping with narrow peak regions of Yrr1 ChIP were compared to those of all the loci in the RNA-Seq data (Figure S1 in File S1 and Table S1). A considerable number of Yrr1 peaks identified by CisGenome and MACS2 overlap with highly expressed loci, and are possibly false positives due to hyper-ChIPability. Another reason for concern over false positives is the frequent occurrence of negative peaks (enrichment in input over ChIP). Given the concerns, an extra screening procedure is necessary to exclude false-positive peaks from downstream analyses. Therefore, an *in silico* method was developed in this study in an attempt to identify high-confidence peaks.

Based on their shape metrics, three high-confidence regions were identified in the ChIP data set as binding sites for Yrr1 alleles (Subsection “Determine the confidence in ChIP-Seq peaks based on peak shape metrics,” Figures S2–S4, and Tables S5–S7 in File S1, Table S3, and Table S4). These three ChIP regions were represented using ChIP minus input (Figure S1 in File S1) and log_2_(fold change) (Figure S4 in File S1). Two DNA motifs at potential Yrr1-binding sites were predicted using the three high-confidence regions (Figure S5 in File S1 and Table 2). Motif 1 shared the consensus sequence “CGGA” (or “TCCG” as reverse complement) with potential Yrr1-binding motifs identified in previous studies (Le Crom *et al.* 2002; Morozov and Siggia 2007; Badis *et al.* 2008; Zhao *et al.* 2009; Zhu *et al.* 2009; de Boer and Hughes 2011). Motif 2 did not share a consensus sequence with any previously reported Yrr1-binding motif.

**Table 2:** Three consolidated peak regions with highest confidence of containing functional binding sites of Yrr1

Consolidated Peak Region			Motif 1		Motif 2	
Identifier	Chromosome	Coordinates	Sequence	Coordinates (Strand)	Sequence	Coordinates (Strand)
201	IV	454940–465905	TAAACGGAAATGGG	465396–465409 (−)	ATATAAAACAAAT	465506–465518 (+)
659	X	606408–607949	TCATCGGAATTGAG	607293–607306 (+)	AAATACGCGGAAT	607347–607359 (+)
527	VII	893849–894948	TACACGGAAATAGG	894482–894495 (+)	AAATAACGAAAAT	894586–894598 (+)

### Correlation between high-confidence ChIP peaks and the expression of their nearby genes in 4NQO

**Figure 2:**
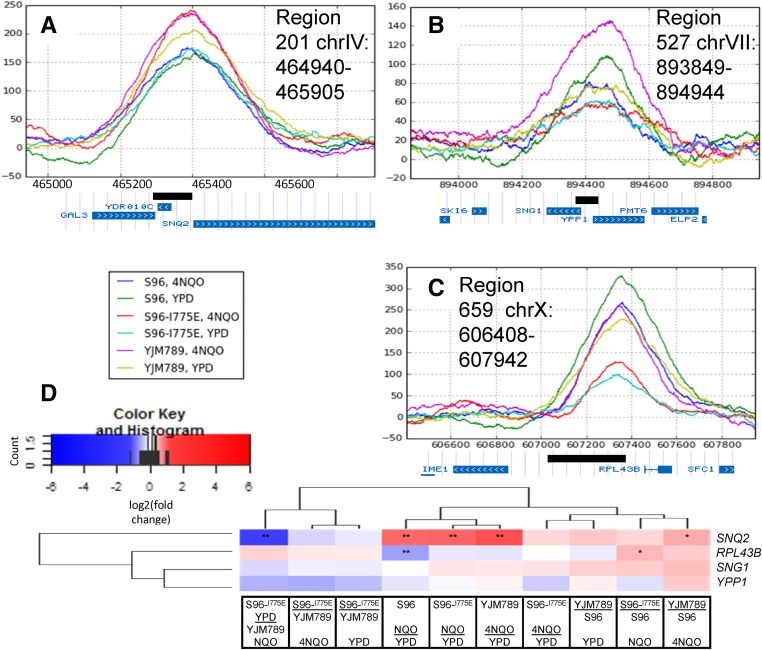
ChIP peaks shown as ChIP minus input within the three high-confidence regions and corresponding mRNA expression. (A) *SNQ2* (peak 201), (B) *SNG1* and *YPP1* (peak 659), and (C) *RPL36B* (peak 527). The plotted data were mean pileup values of normalized ChIP minus input generated by MACS2, using two model-building options for three biological replicates per combination of the *YRR1* allele and growth medium (*Materials and Methods*). Each plotted region is represented as a black box together with nearby genes shown in the University of California, Santa Cruz Genome Browser (http://genome.ucsc.edu) (Kent *et al.* 2002). (D) Heatmap showing differential RNA expression for genes *SNQ2*, *RPL43B*, *SNG1*, and *YPP1* near the three regions (201, 659, and 527) with high confidence of containing functional binding sites for Yrr1, among different conditions. Log_2_(fold change of FPKM) values calculated by DESeq2 are represented in blue and red. Significant differential expression instances are highlighted based on *q*-values, where “*” represents between 0.005 and 0.05, and “**” for < 0.005. ChIP, chromatin immunoprecipitation; Chr, chromosome; FPKM, fragments per kilobase gene per million mapped fragments.

Regulation of genes by the binding of a transcription factor is assumed by the mere presence of the transcription factor. The expression of genes downstream of Yrr1 was further investigated. Pearson’s test was performed to investigate the correlation between the ChIP peak strengths in the three high-confidence regions (201, 659, and 527) and the expression levels (represented by FPKM values) of their nearby genes *SNQ2*, *RPL43B*, *SNG1*, and *YPP1* (Table 3 and Table 4). Expression of *SNQ2* (FPKM values) increased significantly, and Yrr1 binding upstream of *SNQ2* (summit height of ChIP peaks in region 201) increased in response to 4NQO for all the three alleles Yrr1^S^, Yrr1^IE^, and Yrr1^Y^ (Figure 2A, Table 3, and Table S1). In addition, *SNQ2* expression in 4NQO was significantly higher for Yrr1^Y^ than for Yrr1^S^, but less when comparing yeast grown in YPD with the Yrr1^IE^ allele to yeast grown in 4NQO with the Yrr1^Y^ allele (Figure 2D, Table 3, and Table S1). This indicated that the I775E mutation was not sufficient to maximize the expression of *SNQ2* on its own. Although the Yrr1^IE^ allele could phenocopy Yrr1^Y^ in response to 4NQO, the mutated allele was not the same as the Yrr1^Y^ or Yrr1^S^ allele. There were other variable residues in Yrr1 that were subject to regulation in response to 4NQO, as indicated by the expression of *SNQ2* by the Yrr1^Y^ allele in 4NQO being higher than Yrr1^IE^ in YPD.

**Table 3:** ChIP peak metrics of the three high-confidence regions and expression of their downstream genes for different conditions (combinations of Yrr1 allele and growth medium)

Consolidated Peak Region ID	Value Type	S96, 4NQO	S96, YPD	S96-I775E, 4NQO	S96-I775E, YPD	YJM789, 4NQO	YJM789, YPD
201	Summit height*^a^*	177.3	167.7	242.6	177.2	238.7	208.8
	Pileup log2fc*^a^*	0.900	0.756	1.084	0.991	1.012	1.167
	*SNQ2* FPKM*^b^*	144.5	64.8	177.6	77.8	199.0	83.5
659	Summit height	267.8	330.4	132.0	100.6	260.2	232.0
	Pileup log2fc	1.542	1.940	0.751	0.694	1.308	1.610
	*RPL43B* FPKM	1599	2071	2254	2143	1914	1931
527	Summit height	84.47	111.6	65.97	65.53	150.4	82.12
	Pileup log2fc	0.509	0.553	0.582	0.570	1.042	0.556
	*SNG1* FPKM	27.0	22.8	34.5	26.6	34.2	28.3
	*YPP1* FPKM	37.8	36.8	33.8	29.8	45.6	39.2

a Summit height and pileup log2fc: mean summit height and mean pileup log_2_(fold change) of three biologcial replicates using two model building options, respectively (*Materials and Methods*). ID, identifier; 4NQO, 4-nitroquinoline-1-oxide; YPD, Yeast Peptone Dextrose.

b FPKM: mean fragments per kilobase of transcript length per million mapped reads of two biological replicates, determined from Rsubread counts of reads.

**Table 4:** Pearson’s correlation test results on ChIP peak metrics of the three high-confidence regions and expression of their downstream genes for different combinations of *Yrr1* allele and growth medium

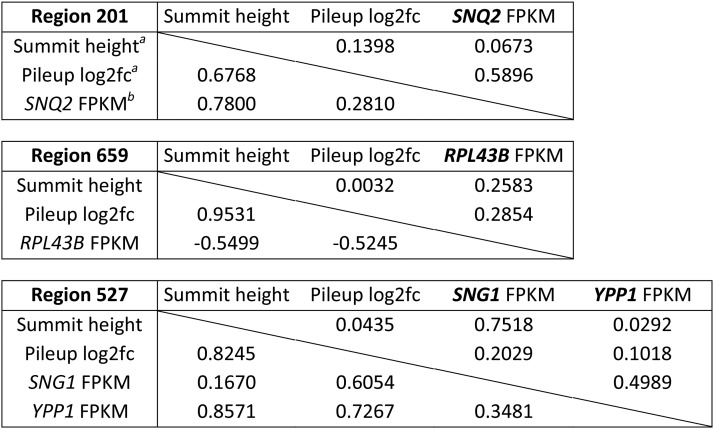

a Summit height and pileup log2fc: mean summit height and mean pileup log_2_(fold change) of three biological replicates using two model building options (Materials and Methods)

b FPKM: mean Fragments Per Kilobase of transcript length per Million mapped reads of two biological replicates, determined from Rsubread counts of reads

ChIP region 527 is located upstream of two divergent genes, *SNG1* and *YPP1*. The protein structure of Sng1 has not yet been well characterized, but transformation of *SNG1* into *S. cerevisiae* via a multicopy vector conferred resistance to 4NQO (Servos *et al.* 1993). In addition, a gain-of-function Yrr1 mutant increased expression of *SNG1*, and Yrr1 directly binds the *SNG1* promoter *in vitro* (Le Crom *et al.* 2002). Our data connected the contribution of *SNG1* to 4NQO resistance and the regulation of *SNG1* by Yrr1 by showing increased Yrr1 ChIP peak strength in region 527. The correlation between Yrr1 ChIP peak strength (Table 3) and *SNG1* expression was positive but weak in this study (Table 4). The other gene for which ChIP region 527 is upstream, *YPP1*, is essential to *S. cerevisiae* and encodes a vesicle-trafficking protein involved in vacuole-targeted endocytosis (Flower *et al.* 2007). *YPP1* has not been reported to contribute to 4NQO responses or to be regulated by Yrr1. There were no significant changes of *YPP1* expression in response to 4NQO or among alleles (Figure 2D).

ChIP region 659 is located upstream of *RPL43B*, which encodes L43B, a protein of the ribosomal 60S subunit. Yrr1^S^ bound the strongest in YPD upstream of *RPL43B* and Yrr1^IE^ bound the weakest in YPD (Figure 2C). The Yrr1^S^ binding decreased in 4NQO compared to YPD, while Yrr1^IE^ binding decreased in YPD compared to 4NQO. The binding of Yrr1^IE^ was lowest of all the alleles. There was a modest binding increase when yeast were grown in 4NQO. Yrr1 is not known to regulate *RPL43B*. RNA levels of *RPL43B* decreased modestly in response to 4NQO for all three alleles (Figure 2D and Table 3). When all the conditions were included in one test, weak negative correlation was observed with binding and expression; however, the expression of *RPL43B* aligned with the comparisons of the growth of yeast in different conditions (Table 4). For example, growth of yeast with Yrr1^S^ was strongly inhibited in 4NQO compared to YPD (Gallagher *et al.* 2014). It should be noted that expression of *RPL43B* decreased the most when Yrr1^S^ was expressed in yeast grown in 4NQO compared to YPD (Figure 2D).

### Impact of overexpression of differentially expressed genes on yeast growth in 4NQO

**Figure 3:**
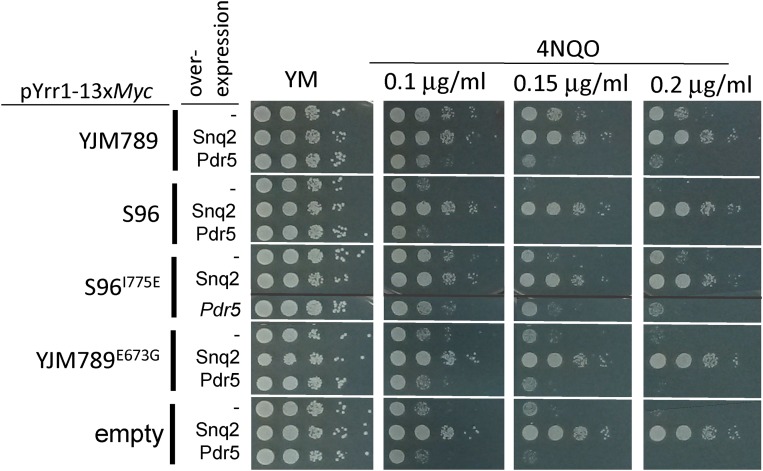
Growth assays of yeast overexpressing ABC transporters. S288c (FY3 *yrr1*Δ) yeast with different alleles of Yrr1 were transformed with either an empty plasmid (-), or a plasmid overexpressing *SNQ2* or *PDR5*. Ten-fold serial dilutions of yeast grown in selective media to maintain both plasmids were spotted onto increasing amounts of 4NQO. Plates were incubated for 3 d and photographed. 4NQO, 4-nitroquinoline-1-oxide; ABC, ATP-binding cassette; YM, Yeast Minimal.

Several genes showed increased expression levels in yeast expressing Yrr1^Y^ when grown in 4NQO. To assess whether overexpression can bypass the Yrr1^S^ 4NQO sensitivity, genes from Figure 1A were overexpressed in yeast along with the different alleles of Yrr1 or a *yrr1* knockout. Overexpression plasmids encoding *SNQ2* and the related ABC transporter *PDR5* were transformed into yeast carrying four alleles of Yrr1 including Yrr1^EG^, which is 4NQO-sensitive. In every instance, *SNQ2*, but not *PDR5*, overexpression could rescue growth in the presence of 4NQO, even more than yeast carrying Yrr1 4NQO-resistant alleles (Figure 3). A selection of diverse genes was placed under a *GAL* promoter to drive overexpression, but only *SNQ2* overexpression could rescue yeast growth on 4NQO (Figure S9 in File S1). While this does not rule out other proteins that may contribute to the 4NQO response, this indicates that higher *SNQ2* expression in 4NQO is the major contributor to higher resistance to 4NQO. The effect of the T775 site in Yrr1 was not sufficient to be the sole regulator of a well-known target of regulation such as *SNQ2*, in contrast to the growth of yeast in different conditions. Transcriptomics and ChIP-Seq are more quantitative and show that the regulation is much more complicated than a simple growth assay previously revealed.

### Impact of Yrr1 on phenotypic expression in nonfermentable carbon sources

**Figure 4:**
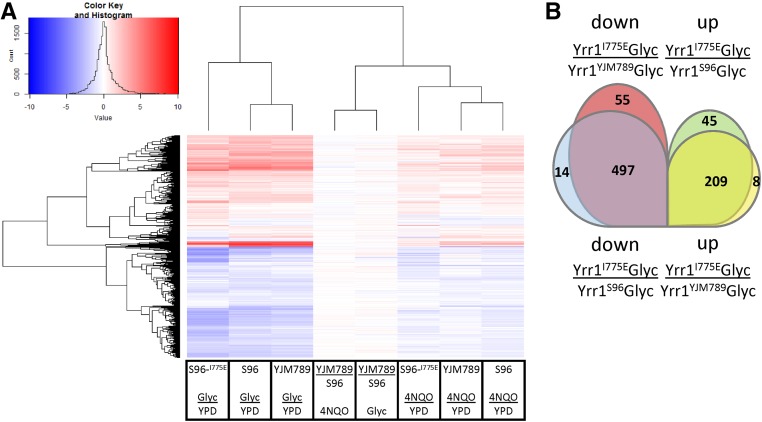
Venn diagrams of mRNAs that increase and decrease between strains with different Yrr1 alleles grown in YPD or Glyc. (A) Expression of genes that decrease when cells are shifted to Glyc with different alleles of Yrr1. (B) Comparisons of genes that decrease or increased between yeast carrying different alleles of Yrr1 when grown in glycerol. 4NQO, 4-nitroquinoline-1-oxide; Glyc, glycerol; YPD, Yeast Peptone Dextrose medium.

4NQO resistance is linked to poor growth in nonfermentable carbon sources (Gallagher *et al.* 2014). To metabolize carbon sources such as these, yeast shift from fermentation to respiration in the mitochondria. To understand how alleles of Yrr1 change gene expression when yeast are grown in media that requires respiration, mRNA levels were compared between yeast with Yrr1^Y^, Yrr1^S^, or Yrr1^IE^ alleles (Table S8). Overall levels of mRNAs from yeast expressing Yrr1^Y^ and Yrr1^S^ were more similar to each other than mRNAs from yeast expressing Yrr1^IE^ when shifted to glycerol (Figure 4, A and B). Between Yrr1^Y^ and Yrr1 ^S^ yeast, 18 mRNAs were downregulated in glycerol, including *ATO3*, and 87 genes were upregulated, including *AAH1* and a subset of components of the ETC (*COX5A*, *COX5B*, *COX6*, *COX8*, *COX9*). Yrr1^IE^ mRNA expression was very different when comparing Yrr1^Y^ and Yrr1^S^ yeast, with 497 genes decreased in expression, including ribosomal protein-encoding genes, ribosome biogenesis genes, and ETC and ATPase genes, as compared to z-score of log2(fold) change in expression comparing mRNA expression between yeast carrying different alleles of Yrr1 grown in YPglyc (Table S8). Only 14 genes decreased in the comparison between Yrr1^S^ and Yrr1^IE^, and 55 genes were downregulated specifically in Yrr1^IE^ compared to Yrr1^Y^.

When comparing mRNAs across strains grown in glycerol, Yrr1^S^ and Yrr1^Y^ were quite similar. The expression of 418 genes that increased in unison across strains was independent of differences between Yrr1 alleles, including mRNAs encoding components of the ATPase (*ATP1*, *ATP2*, *ATP3*, *ATP4*, *ATP5*, *ATP7*, *ATP10*, *ATP15*, *ATP16*, *ATP17* and *TIM11*) and ETC [*COR1*, *CYT1*, (*QCR2*, *QCR7*, *QCR8*, *QCR9*, *QCR10*), *RIP1*, *SDH1*, *SDH2*, *SDH3*, *SDH4*, *SHH4*, and *COX1*, *COX3*, *COX4*, *COX5A*, *COX6* and *COX15*.]. In comparing the Yrr1^IE^ allele to the wild-type alleles grown in glycerol, many genes were upregulated in the mutant. Nonetheless, Yrr1^IE^ was more similar to Yrr1^Y^ than Yrr1^S^ among genes that were upregulated. The 209 shared genes that were upregulated in the mutant had diverse functions. In a similar analysis of downregulated genes in the mutant compared to the wild-type alleles, 497 genes of diverse functions were downregulated Yrr1^IE^. However, Yrr1^IE^ was more similar to Yrr1^S^ than Yrr1^Y^ when comparing downregulated genes.

Direct comparisons of transcription and translation between S288c (specifically S96) and YJM789 yeast have consistently found increased expression of YJM789 mRNAs encoding genes involved in respiration (Sun *et al.* 2016), and relative protein levels between the two strains were also higher when YJM789 yeast were grown in the presence of dextrose (Rong-Mullins *et al.* 2017). These lines of evidence indicate that YJM789 yeast constitutively upregulates respiration mRNAs and proteins in comparison to S96, regardless of metabolic state, even when cells are grown in dextrose. YJM789 yeast grow slower on nonfermentable carbon sources (Gallagher *et al.* 2014) and have a lower electron gradient across the mitochondrial membrane than S96 yeast (discussed below). *Cox5B* expression occurs during anaerobic growth (hypoxic), as compared to its paralog *Cox5A*, which is expressed during aerobic growth (Burke *et al.* 1997). *COX5B* was increased in yeast expressing Yrr1^IE^ and Yrr1^Y^ compared to Yrr1^S^ when grown in glycerol, while *COX5A* was decreased between Yrr1^IE^ when compared to Yrr1^S^. Over half of the 452 genes that were downregulated in response to growth on glycerol were involved in ribosome biogenesis, structural ribosomal proteins, and translation (Warner 1999; Woolford and Baserga 2013). Ribosomal mRNAs decreased the most between yeast expressing Yrr1^Y^, which grow poorly in this condition. Ribosome biogenesis is a costly process, and it is quickly reduced when yeast growth slows (Warner 1999; Woolford and Baserga 2013).

### Expression of purine de novo biosynthetic pathway during respiration

Another mRNA that was differentially expressed in yeast expressing Yrr1^IE^ compared to Yrr1^S^ and Yrr1^Y^ was *AAH1*. Expression of *AAH1* in Yrr1^IE^ yeast grown in glycerol compared to YPD decreased 4 log_2_(fold) compared to yeast expressing other alleles [log_2_(fold) decrease]. Aah1 is an enzyme that converts adenine to hypoxathanine in the purine salvage pathway (Woods *et al.* 1984). While all strains irrespective of the allele of Yrr1 downregulated many genes in the purine *de novo* biosynthetic pathway in both 4NQO and glycerol, these yeast also downregulated *IMD3*, while *IMD4* increased, in glycerol, genes which encode other proteins in the purine salvage pathway (Table S1 and Table S8). Evidence from other systems suggests that the purine salvage pathway can generate purine-based antioxidants in the mitochondria (Becker 1993; Kristal *et al.* 1999). Respiration is the primary source of endogenous ROS in yeast, and catalase (*CTT1*) and mitochondrial superoxide dismutase (*SOD2*) levels were also increased in yeast grown in glycerol.

Shifting yeast to different environmental conditions changed the expression of hundreds of mRNAs. To gain perspective on the role of different alleles of Yrr1, GO term analysis was carried out (Table S9). Changes for each allele were compared between yeast grown in 4NQO or glycerol, and compared to the yeast carrying the same allele of Yrr1 grown in YPD. Representative pathways were graphically represented using the −log of the *p*-value. Yeast containing Yrr1^IE^ had the fewest GO terms change in either condition (Figure S10 in File S1). Because the Yrr1^IE^ allele is thought to be in part a constitutively active allele, these changes may reflect the phosphorylated state of Yrr1^Y^. Pathways required for active growth, such as ribosome biogenesis, were consistently downregulated (by strain-specific extents). Yrr1^IE^ downregulated a similar number of genes in the ribosome biogenesis pathway across both conditions, while Yrr1^Y^ showed less downregulation of this pathway by comparing the number of genes in each GO term. Purine biosynthesis, amino acid activation, and IMP biosynthesis were more downregulated in Yrr1^Y^ compared to Yrr1^S^, while Yrr1^IE^ showed no change. There were also predictive patterns in pathways that were upregulated compared to YPD. By comparing the number of genes in each GO term, yeast carrying the Yrr1^S^ allele had more genes involved in cellular respiration upregulated in both 4NQO and glycerol than Yrr1^Y^ and Yrr1^IE^.

### Yrr1 regulation of metabolism and growth under nonfermentable carbon sources

**Figure 5:**
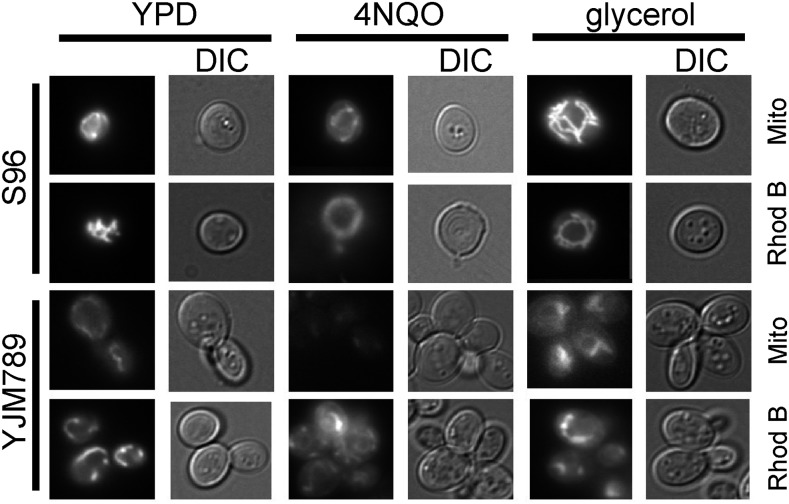
Live mitochondrial staining of S96 and YJM789 yeast grown in YPD, 4NQO, and glycerol with Mitotracker and Rhodamine B hexyl ester. 4NQO, 4-nitroquinoline-1-oxide; DIC, differential interference contrast; YPD, Yeast Peptone Dextrose medium.

Because of the differences in the growth of YJM789 and S96 in glycerol, we assessed the function of the mitochondria from both strains. Glycerol is a nonfermentable carbon source that requires respiration by the mitochondria to metabolize the glycerol. We examined the function of mitochondria using vital stains. Mitotracker stains the mitochondria based on the electron potential across the membrane, and mitochondria from YJM789 were lightly stained compared to those from S96 (Figure 5). In contrast, Rhodamine B hexyl ester, which stains the lipids of the mitochondria, equally stained YJM789 and S96. Taken together with the altered growth on glycerol, this suggested that YJM789 cells have less oxidative phosphorylation and consequently less respiration. The mitochondrial DNA contents of these cells are the same, and although there are SNPs between these strains in the mitochondrial genome, swapping the mitochondria does not change growth on glycerol (Sun *et al.* 2016).

**Figure 6:**
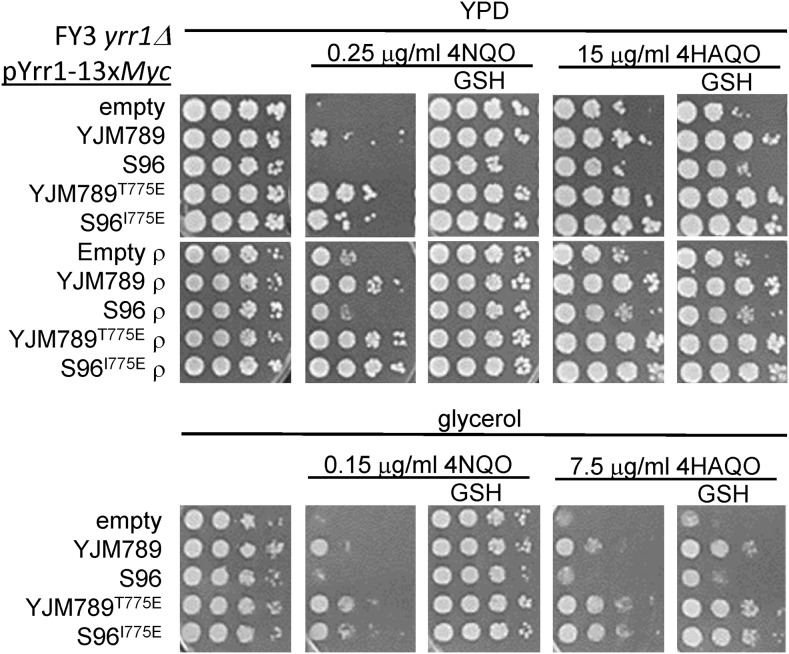
Growth assays of grande and petite (ρ) yeast (FY3 *yrr1*::*URA3*) expressing different alleles of Yrr1 on different combinations of carbon source and derivatives of 4NQO. All yeast contained the pYrr1-13x*Myc* plasmid, and strain labels indicate the allele of *YRR1* encoded on the plasmid. Ten-fold serial dilutions of FY3 *yrr1*Δ yeast grown in YPD or glycerol media with 4NQO or 4HAQO supplemented with the antioxidants GSH. Plates were incubated for 3 d and photographed. 4HAQO, 4-hydroxyaminoquinoline-1-oxide; 4NQO, 4-nitroquinoline-1-oxide; GSH, glutathione; YPD, Yeast Peptone Dextrose medium.

The ability to respire might also affect the 4NQO toxicity. We tested the 4NQO response in cells that contain mitochondria that cannot respire (petite) by removing the mitochondrial genome. Strains were made respiration deficient (ρ^0^) and, when plated on 4NQO, they grew better than grande yeast (ρ^+^), which were respiration-proficient strains (Figure 6, top row). Here, we tested both wild-type alleles of Yrr1, Yrr1^IE^, and Yrr1^TE^ (T775E mutation in the Yrr1 allele from YJM789). We found that more 4HAQO was required to slow the growth of such yeast, which was consistent with our hypothesis that 4NQO toxicity was related to its metabolism and the resultant production of ROS. 4NQO toxicity was indeed mediated through the mitochondria, because the resistance of yeast to 4NQO was decreased in the nonfermentable carbon source where respiration was required (Figure 6, bottom row). To confirm that respiration was generating free radicals from 4NQO metabolism, the antioxidant glutathione (GSH) rescued the enhanced 4NQO toxicity in glycerol media (Figure 6, bottom row). The potentiation of 4NQO generated free radicals that were quenched by the addition of GSH. 4HAQO toxicity was not rescued by antioxidants, further supporting the importance of respiration-induced ROS in 4NQO toxicity. Consistent with the parent strains, the *yrr1*Δ yeast expressing mutant alleles of Yrr1 with glutamic acid at 775 were more resistant than wild-type parental alleles (Figure 6). All strains could be rescued from 4NQO by GSH in YPD and were more sensitive to 4NQO when grown on glycerol. Isogenic yeast expressing different alleles were also sensitive to 4HAQO, which could not be rescued by the addition of GSH. Previous studies in cell lysates have found that 4NQO conversion to 4HAQO is stimulated by oxygen, and that metabolic activation increases when reducing compounds are available (Blaglow *et al.* 1977). In human cell culture, adding GSH increases the viability of cells treated with 4NQO and decreases the production of 4HAQO (Arima *et al.* 2006). Altering levels in the purine salvage pathway may also change the metabolism of 4NQO, as 4HAQO-purine adducts form (Kitani *et al.* 1983; Loret *et al.* 2007). Yrr1 has additional roles to play in the 4NQO response because the *yrr1*Δ yeast from both resistant and sensitive yeast has hindered growth compared to the respective wild-type parents.

**Figure 7:**
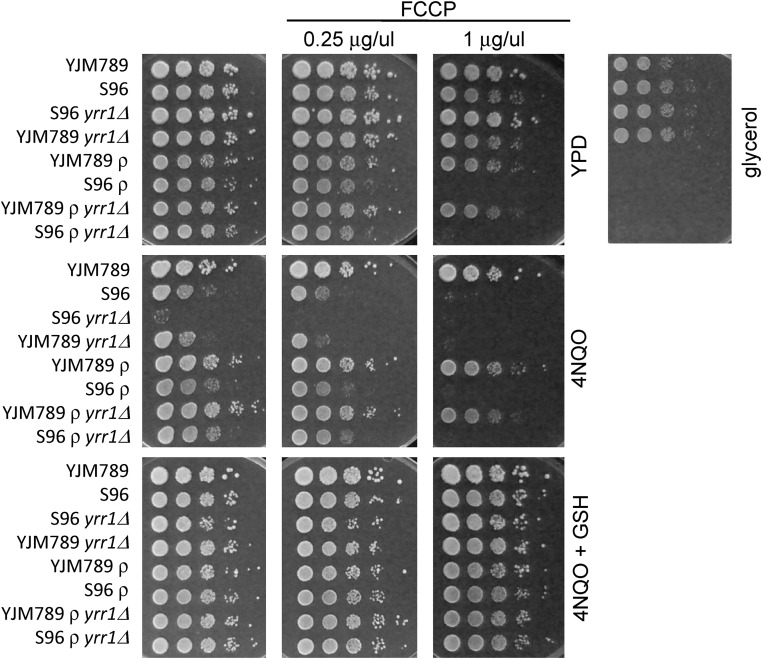
Growth assays of YJM789 or S96 yeast and different combinations of wild-type or *yrr1*Δ mutants, and grande or petite (ρ) with FCCP. Ten-fold serial dilutions of yeast grown YPD with 4NQO supplemented with antioxidants GSH. Plates were incubated for 3 d and photographed. 4NQO, 4-nitroquinoline-1-oxide; FCCP, Carbonyl cyanide-*4*-(trifluoromethoxy)phenylhydrazone; GSH, glutathione; YPD, Yeast Peptone Dextrose medium

To pinpoint which stage of the ETC may be working to increase 4NQO toxicity, drugs that block specific steps were tested in conjunction with 4NQO. Concentrations of drugs that interfere at various steps of respiration were optimized to inhibit growth on glycerol, yet permit growth on YPD. In this case, when grown in the presence of these drugs, yeast were unable to respire and were functionally petite, but the ETC was stopped at different steps based on the target of each drug. If these chemical respiration blockers work at the same step as 4NQO, then no decreased growth inhibition would be seen. Antimycin A blocks the transfer of electrons between cytochrome b and cytochrome c, and decreases oxygen consumption, which occurs at complex IV. Myxothiazol inhibits cytochrome bc1 by competing with ubiquinol, and the effect of myxothiazol binding induces a red-shift to the visible absorption spectrum of reduced heme bl. Oligomycin A is an inhibitor of ATP synthase and blocks proton flow. FCCP breaks the proton gradient because it permeablizes the mitochondrial membrane and allows electrons to flow around the ATP synthase. Because this occurs after Complex IV and possibly as the cells try to compensate for decreased ATP, the oxygen consumption of FCCP-treated cells increases (Schnellmann 2013). There was no change in growth with antimycin A, myxothiazol, or oligomycin A with 4NQO, but FCCP exacerbated sensitivity to 4NQO (Figure 7). Blocking the ETC causes electrons to back up, which we posit radicalizes oxygen, increasing the damage from 4NQO treatment. Nevertheless, the addition of GSH rescues all growth inhibition.

### Gene expression patterns in 4NQO and nonfermentable carbon sources

In the presence of 4NQO, the expression of *SNQ2* was significantly higher in yeast with the Yrr1^Y^ allele than with the Yrr1^S^ allele. For all three alleles, *SNQ2* expression was not significantly lower when the yeast cells utilized glycerol when compared to yeast carrying those alleles grown with dextrose as the carbon source. Because cells containing Yrr1^Y^ grow better than those containing Yrr1^S^ in 4NQO (but grew less in YPglyc media), higher expression of *SNQ2* can be associated with higher resistance to 4NQO and with inhibited growth in glycerol media.

### Conclusions

**Figure 8:**
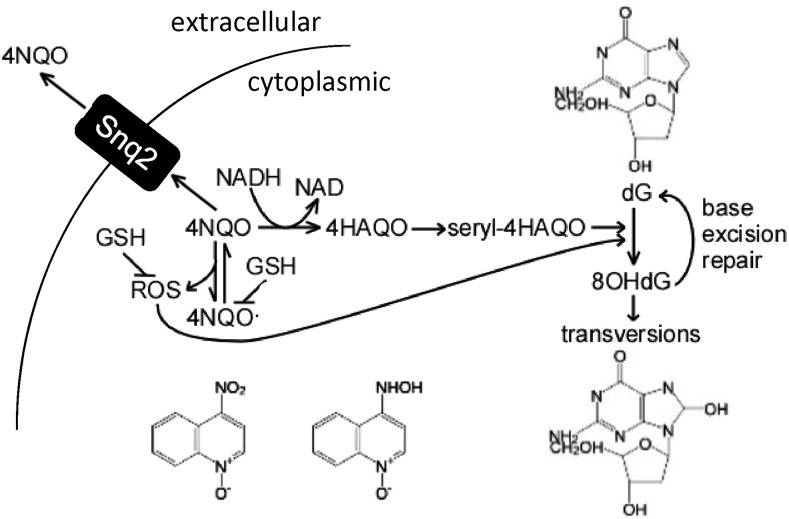
Model 4NQO metabolism in yeast. 4QNO is reduced into 4HAQO while also generating ROS. The ROS byproducts of 4NQO metabolism can be quenched by GSH. Metabolism of 4HAQO does not produce ROS, and so 4HAQO growth inhibition could be mitigated by the addition of GSH. 4HAQO is conjugated to a seryl and oxidized guanine (dG) into 8OHdG. Oxidized nucleotides in DNA are typically repaired by base excision. Snq2 localized to the cell membrane exports 4NQO. Increased expression of Snq2 decreases the growth inhibition by 4NQO. 4HAQO, 4-hydroxyaminoquinoline-1-oxide; 4NQO, 4-nitroquinoline-1-oxide; 8OHdG, 8-Oxo-2’-deoxyguanosine; GSH, glutathione; ROS, reactive oxygen species.

The role of Yrr1 in 4NQO toxicity may represent the connection between the cell’s responses to various sources of stress, such as nutrient availability (carbon source) and xenobiotics (4NQO), leading us to propose a metabolic model for 4NQO mechanism (Figure 8). In yeast that actively respire, 4NQO is more toxic because of the production of both 4HAQO and ROS, and growth can be rescued by the addition of GSH, which quenches the ROS but not the 4HAQO effects. GSH reduces ROS, and can be directly conjugated to 4NQO products (Peklak-Scott *et al.* 2005) and then transported out of the cell by Snq2. The addition of 20 times more 4HAQO was required to inhibit yeast growth to the same extent as 4NQO, so most of 4NQO’s toxicity can be traced to the process of conversion, not the 4HAQO metabolite itself. Petite yeast cannot respire and hence were more resistant to 4NQO because 4NQO was not converted. Conversely, forcing yeast to respire increased the toxicity of 4NQO. The unexpected relative resistance to 4NQO of petite yeast illustrated that shifting the carbon metabolism and by proxy the redox state of yeast altered 4NQO response. In other words, repressing respiration increased 4NQO resistance, whether that was by actively preventing respiration in petite cells or expressing alleles of Yrr1 that cause yeast to respire less. Because petite 4NQO-resistant yeast could not grow on a nonfermentable carbon source, it is a most extreme example of reduced growth on a nonfermentable carbon source compared to the only slowed growth of Yrr1^Y^-carrying yeast in similar conditions. The variation in gene expression among yeast with different Yrr1 alleles was a combination of direct regulation and indirect change. The indirect change in gene expression involved general pathways, which were downregulated in response to stress.

In an effort to correlate ChIP-Seq and transcriptomics in a variety of environmental conditions, we assessed changes in gene expression induced by different alleles of a single transcription factor. While a single polymorphism can flip a 4NQO-sensitive allele to a 4NQO-resistant allele by inducing the transcription of Snq2, the simplicity of this model did not translate well to the growth of yeast in glycerol. The expression of Yrr1^Y^ conferred a productive transcriptional response to 4NQO, but slowed the growth of yeast in nonfermentable carbon sources. The Yrr1^IE^ allele increased the 4NQO resistance more than either wild-type allele; however, examination of the transcriptomic data revealed significant differences in genome-wide expression between the Yrr1^IE^ and Yrr1^Y^ alleles, despite similar phenotypes in 4NQO. There are likely additional modes of Yrr1 regulation, in particular when cells are grown in glycerol, which precluded the correlation of ChIP-Seq of a single transcription factor and changes in the transcriptome. The changes in the transcriptome between three different conditions with the different alleles of Yrr1 combine not only inputs from Yrr1, but also regulators that respond to these conditions. The transcriptomic data set produced here can be further used for the analysis of expression variation in different carbon sources due to genetic variation in a single transcription factor.

## 

## Supplementary Material

Supplemental material is available online at www.g3journal.org/lookup/suppl/doi:10.1534/g3.117.300138/-/DC1.
