# Supplementary material for: Transcriptional Profiling of Saccharomyces cerevisiae Reveals the Impact of Variation of a Single Transcription Factor on Differential Gene Expression in 4NQO, Fermentable, and Nonfermentable Carbon Sources
Source: G3 (Bethesda). 2017 Dec 5;8(2):607–19. doi: 10.1534/g3.117.300138 (PMC5919752; doi:10.1534/g3.117.300138)
Supplement: Supplementary file 7 [file 607FileS1.docx]

# Supporting text Rong-Mullins 2017

**Determine confidence in ChIP-Seq peaks based on peak shape metrics**

The loci showing zero-FPKM by Rsubread under all the tested conditions (combinations of Yrr1 allele and growth medium) were not considered because they were probably either (1) rRNA or tRNA genes whose reads were filtered out for RNA-Seq to reduce bias in FPKM calculation or (2) dubious ORFs unlikely to encode functional proteins. After this exclusion, 6620 loci showed nonzero FPKM values under at least one condition, and 933 loci overlapped with 888 narrow peak regions of the Yrr1 ChIP. The modes of the FPKM distributions of all the 6620 loci were < 2^5^ (32) (Figure S1C). In contrast, the modes of loci overlapping with peak regions shifted up between 2^5^ (32) and 2^10^ (1024) had modes around 2^10^ (depending on condition). There were 819 consolidated peak regions containing negative peaks compared to 1136 consolidated peak regions containing positive peaks (enrichment in ChIP over input). Thus, there may be a considerable number of false positive peaks that occurred from the same causes as negative peaks (i.e. uneven sequencing coverage unrelated to ChIP enrichment).

Previously ChIP-seq was acquired and reanalyzed [1]. The bedGraph pileup data generated by MACS2 via option --bdg was used to examine the shapes of peaks identified by CisGenome and MACS2. The pileup data was normalized so that every sample, whether ChIP or input, had a genome-wide sum pileup of 7.5^e^8, approximately equivalent to a mean coverage of 62.5X when the genome size is assumed to be 12^e^6 bp. The normalized pileup values of input were subtracted from those of ChIP for the same condition (combination of *YRR1* allele and growth medium) at each nucleotide position, to generate the raw ‘ChIP minus input’ (**Cmi**) pileup data. Rolling means in windows of 200 bp were calculated for each raw Cmi pileup dataset. Only the pileup data within the consolidated peak regions (1136 in total, see manuscript Materials and Methods for how they were generated) was used in the following procedure. For each consolidated peak region, the chromosomal coordinates (X_i_) were found corresponding to the following extrema (Y_i_) on the rolling mean curve of Cmi: the global maximum (X_gmax_ corresponding to Y_gmax_), then the local minima (X_lmin1_ and X_lmin2_ respectively corresponding to Y_lmin1_ and Y_lmin2_) and the local maximum (X_lmax_ corresponding to Y_lmax_) closest to X_gmax_ but at least 20 bp away from X_gmax_ (Figure S2 A, D). In raw Cmi (not rolling mean), the global maximum pileup value (Y_gmax_raw_) was termed **summit height**; the pileup value at X_lmax_ was termed **2^nd^ summit height**, then the difference between summit height and 2^nd^ summit height was termed **rise from 2^nd^ summit**; the mean pileup value throughout the peak region excluding the region between X_lmin1_ and X_lmin2_ was termed **background mean**; then the difference between summit height and background mean was termed **rise from background mean** (Figure S2 B, E). The normalized pileup values of ChIP and input were also plotted as two curves in the same graph (Figure S2 C, F); the difference between the global maximal pileup values of ChIP and input curve was termed **summit difference**; the difference between the chromosomal coordinates of the two maxima was termed **summit distance**. The peak region initially identified by MACS2 for the respective sample (not yet consolidated with overlapping peaks in other samples) was shown as **MACS2 peak region** (Figure S2 C, F); peaks identified by CisGenome were not shown because the pileup data used here was generated by MACS2 instead of CisGenome.

The term **peak instance** means the occurrence of a peak within a certain consolidated peak region in a pileup dataset (for how consolidated peak regions were generated, see manuscript Materials and Methods). A pileup dataset represents a combination of Yrr1 allele, growth medium, replicate and peak-calling option. There were three alleles (Yrr1^S^, Yrr1^Y^ and Yrr1^IE^) and two growth media (YPD without 4NQO and YPD with 4NQO), therefore six conditions; there are three replicates for each condition; for each replicate, peak calling by MACS2 was done using two options (-m 2,30 and --nomodel). In total there are 36 pileup datasets for the ChIP-Seq experiment thus 36 peak instances for each consolidated peak region.

For each consolidated peak region, the mean X_lmin1_ and X_lmin2_ values of all the applicable peak instances were respectively used as the **left** and **right bounds** of this consolidated peak region. The region between the left and right bounds is termed the **narrow peak region** of the consolidated peak region. When a left or right bound is not available (no X_lmin1_ or X_lmin2_ was available for any peak instance), the start or end of the consolidated peak region is used as the left or right bound, respectively, to delineate the narrow peak region. The length of a narrow peak region is also the length of the peak instance in this consolidated peak region. A narrow peak region is meant to be more focused on the risen (i.e. peak) part of the consolidated peak region and to exclude most of the background parts. Narrow peak regions are primarily used to (1) evaluate overlapping of ChIP peaks with genetic features such as gene or telomere and (2) determine the log_2_(fold change) between ChIP and input using cumulative pileup values (areas under the curves). Inherently, there is one narrow peak region for each consolidated peak region. Two sets of thresholds for peak metrics were used to select peaks with high priority of consideration (Table S6).

There are 8 consolidated peak regions that showed totally 92 peak instances passing threshold Set 1 (Table S7). Regions 201, 659 (e.g. Figure S2) and 527 (e.g. Figure S4) accounted for 66 of those instances, which overlapped with lowly expressed genes (FPKM <= 60) or no genetic feature, therefore are not very likely false positives due to hyper-ChIPability. One other instance was in Region 305 and overlapped with a tRNA gene tS(UGA)I which is probably highly expressed, therefore this instance is likely a hyper-ChIPable false positive. Four consolidated peak regions accounted for the rest of the instances and they all overlapped with telomeres. Telomeres contain repetitive regions where mapping qualities of reads are low, i.e. reads are mapped to multiple regions across the genome. This introduces uncertainty about whether the reads counted as mapped to each telomere region actually originated from this specific region, instead of other regions with similar sequences (probably other telomeres); it also introduces the possibility of inflated read counts over actual local coverage. Therefore, peak instances overlapping with telomeres cannot be confidently considered as true binding sites of Yrr1.

In addition to the 8 regions with instances passing threshold Set 1, 15 consolidated peak regions showed 22 instances that passed threshold Set 2 (Table S7). Region 1064 is upstream of *YRR1* and Yrr1 has been shown to auto-regulate by binding upstream of its own gene (Zhang *et al*. 2001). However, input samples showed pronounced peaks overlapping with peaks in ChIP samples in Region 1064 (Table S4, e.g. Figure S7), which reduced the confidence that the peak instances in Region 1064 are true binding sites instead of sequence enrichment unrelated to Yrr1 binding. The instances in 10 other regions that passed Set 2 overlapped with telomeres or highly expressed genes (FPKM > 100), therefore are likely false positives. The rest 4 regions overlapped with lowly expressed genes or no feature. However, the peak shape metrics in those regions were inconsistent among replicates. Specifically, though one replicate showed a peak instance passing threshold Set 2, at least one other replicate of the same allele, growth medium and peak calling option showed much lower values of shape metrics and much farther from passing threshold Set 2 (Table S4, e.g. Figure S8 A, B, C). In contrast, the peak instances in Region 201, 659 and 527 that passed threshold Set 1 showed much more consistent values among replicates (Table S4, e.g. Figure S8).

Overall, compared to threshold Set 1, the more relaxed Set 2 discovered few additional consolidated peak regions that likely contain true binding sites of Yrr1. It is consequently assumed that further relaxing thresholds are not very likely to discover more potential binding sites with high confidence. Therefore, Regions 201, 659 and 527 are assigned with highest confidence of containing true binding sites. All these three regions were discovered via threshold Set 1 and accounts for majority of the instances passing the thresholds (66 of 92 for Set 1, 77 of 114 for Set 2).

When ChIP and input were flipped to identify regions enriched in input over ChIP (negative peaks), there are 8 consolidated peak regions showing a total of 25 negative peak instances passing threshold Set 1 (Table S4). Since there are 8 consolidated peak regions showing 92 positive peak instances passing threshold Set 1, the false discovery rate for threshold Set 1 was 25 / (92 + 25) = 21.4%. Prior to application of any threshold, there were 4751 positive and 6313 negative peak instances identified by MACS2, giving a false discovery rate of 6313 / (4751 + 6313) = 57.1%. This shows that threshold Set 1 considerably reduced the false discovery rate measured by occurrence of negative peaks. The causes of negative peaks need to be explored to prevent false discovery of protein-DNA interaction based on enrichment in ChIP due to the same causes as enrichment in input. One possible cause is the local biases of sequence coverage introduced during library preparation and sequencing for ChIP-Seq. In addition, the negative peak instances showed different shape characteristics than positive instances in Regions 201, 659 and 527, which can help differentiate between them: (1) some negative instances showed inconsistence occurrence among treatments, i.e. pronounced peaks were only observed in one treatments and hardly in any other treatment, while those positive instances were similarly pronounced across all treatments (Table S4); (2) the other negative instances showed pronounced secondary peaks near the highest peaks within the consolidated peak regions (Table S4, e.g. Figure S3 D, E, F), while there were none to substantially-lower secondary peaks in Regions 201, 659 and 527 (Table S4, e.g. Figure S2, S8).

# Supporting tables

Table S1

**File S1 Rong_2015_SI_tableS1_rna.xlsx**

**Table S1**. Read counts and differential expression of all genes from All RNA-Seq comparing alleles and growth media YPD, 4NQO, YPGlycerol. In each comparison for differential expression in the format of ‘condition/condition_B’, condition_A is the denominator and condition_B is the numerator when calculating fold change. This table contains the following worksheets:

- **Rsubread_count_table**: count table generated by Rsubread package, used for differential expression analysis by DESeq2 package
- **Rsubread_FPKM**: fragment counts per kilobase gene per million mapped fragments (FPKM) data calculated based on ‘**Rsubread_count_table**’
- **DESeq**: differential expression data generated by DESeq2 package

Table S2

**File S2 Rong_2015_SI_tableS2_hm.xlsx**

**Table S2**. Differential expression of genes that were significantly different from RNA-Seq, analyzed using DESeq2 for all the tested loci and comparisons among alleles and growth media YPD, 4NQO. In each comparison ‘condition_A…condition_B’, condition_A is the denominator and condition_B is the numerator when calculating fold change. This table contains the following worksheets:

- **log2fc**: Log_2_(fold change) values. The genes (rows) are arranged according to the heatmap shown in Figure S1.
- **significant**: significant differential gene expression highlighted based on *p*-values adjusted for multi-testing (*q*-values), where ‘*’ represents *q*-value between 0.005 and 0.05, and ‘**’ represents less than 0.005
- **row_dendrogram**: row dendrogram for the heatmap showing clustering patterns of all the tested genes

Table S3

**File S3 Rong_2015_SI_tableS3_chip1.xlsx**

**Table S3**. All the peaks identified by CisGenome and MACS2 from the ChIP-Seq data. This table contains the following worksheets:

- **CisGenome**: positive peaks (enrichment in ChIP over input) identified by CisGenome
- **MACS2_positive**: positive peaks identified by MACS2
- **MACS2_negative**: negative peaks (enrichment in input over ChIP) identified by MACS2

The columns in worksheet **CisGenome** are explained below:

- **MasterID**: simple sequential numeric ID for each identified peak
- **alnshift2bar_prm**: CisGenome parameter -s for function alnshiftbar, two options 0 and 100 were used.
- **seqpeak_prm**: CisGenome parameters –b -w 1 -e 150 -c 3 for function seqpeak, two options 10 and 50 were used for –b.
- **condition** represents combination of allele and growth medium (shown as … in figures):
  - **T1**: Yrr1^Y^, YPD without 4NQO (YJM789, YPD)
  - **T2**: Yrr1^Y^, YPD with 4NQO (YJM789, 4NQO)
  - **T3**: Yrr1^S^, YPD without 4NQO (S96, YPD)
  - **T4**: Yrr1^S^, YPD with 4NQO (S96, 4NQO)
  - **T5**: Yrr1^IE^, YPD without 4NQO (S96-I775E, YPD)
  - **T6**: Yrr1^IE^, YPD with 4NQO (S96-I775E, 4NQO)
- The rest of the columns were directly from CisGenome output, explained in <http://www.biostat.jhsph.edu/~hji/cisgenome/index_files/outputformat_seqpeak.txt>

The columns in worksheets **MACS2_positive** and **MACS2_negative** were directly from MACS2 output, explained in <https://github.com/taoliu/MACS>, except that:

- **m*** in column **name** represents the MACS2 option used to call peaks:
  - **mn**: --nomodel
  - **m2**: -m 2,30

Table S4

**File S4 Rong_2015_SI_tableS4_chip2.xlsx**

**Table S4**. Shape metrics of all the ChIP-Seq peak instances in all the consolidated peak regions in all the pileup dataset. This table contains the following worksheets:

- **mC1_ct_wd_mi_posi_w200c10**: positive peak instances, representing enrichment in ChIP over input
- **mC1_ct_wd_mi_nega_w200c10**: negative peak instances, representing enrichment in input over ChIP

The columns in the table are explained below:

- **op_id** in the format of ‘m*_T*_ChIP*’ (‘*’ means a variable character), represents the identity of a Cmi pileup dataset as well as the ChIP and input datasets associated with it.
  - **m*** represents the MACS2 option used to call peaks:
    - **mn**: --nomodel
    - **m2**: -m 2,30
  - **T*** represents combination of allele and growth medium (shown as … in figures):
    - **T1**: Yrr1^Y^, YPD without 4NQO (YJM789, YPD)
    - **T2**: Yrr1^Y^, YPD with 4NQO (YJM789, 4NQO)
    - **T3**: Yrr1^S^, YPD without 4NQO (S96, YPD)
    - **T4**: Yrr1^S^, YPD with 4NQO (S96, 4NQO)
    - **T5**: Yrr1^IE^, YPD without 4NQO (S96-I775E, YPD)
    - **T6**: Yrr1^IE^, YPD with 4NQO (S96-I775E, 4NQO)
  - **ChIP*** represents one of three replicates, possible values are ChIP1, ChIP2, ChIP3
- **peak_id**: identity of consolidated peak region (please see manuscript Materials and Methods for how consolidated peak regions were generated)
- **chrom**: chromosome
- **chromStart**: start of consolidated peak region
- **chromEnd**: end of consolidated peak region
- **region_length**: length of consolidated peak region
- **cp_Lbound**: X_lmin1_, representing the left bound of a peak instance (narrow peak region)
- **cp_Rbound**: X_lmin2_, representing the right bound of a peak instance (narrow peak region)
- **cp_length**: X_lmin2_ - X_lmin1_, the length of a peak instance (narrow peak region)
- **climaxPos**: chromosome coordinate of the global maximum on raw (not rolling mean) curve of pileup dataset
- **climax_diff**: summit difference
- **climax_dist**: summit distance
- **climax_dV**: summit height
- **background_mean**: background mean
- **c2b_rise**: rise from background mean
- **xtrm_2max_pos**: X_lmax_, chromosome coordinate of the 2^nd^ highest local maximum across the consolidated peak region
- **xtrm_2max_ori_dV**: 2^nd^ summit height
- **c22_rise**: rise from 2^nd^ summit
- **xtrm_1max_pos**: X_gmax_, chromosome coordinate of the global maximum on rolling mean curve of pileup dataset
- **xtrm_1max_ori_dV**: pileup value at X_gmax_

Table S5

**Table S5**. Examples of peak instances with various confidence levels of being high confident binding sites of Yrr1. All peak instances were for allele Yrr1^IE^ and called by MACS2 using option --nomodel; replicate 3 of growth medium YPD with 4NQO (mn_T6_ChIP3) was used for consolidated peak Regions 201 and 659, replicate 2 of growth medium YPD without 4NQO (mn_T5_ChIP2) was used for Regions 25 and 1314. Relatively lower values of shape metrics were highlighted by underlining.

| **Instance ID** | **Obser-vation^a^** | **Consolidated peak region ID** | **MACS2 peak region** | **Shape metrics** | | | | |
| --- | --- | --- | --- | --- | --- | --- | --- | --- |
|  |  |  |  | **Summit height** | **Summit difference** | **Summit distance (bp)** | **Rise from background mean** | **Rise from 2^nd^ summit** |
| 30998 | Upstream of *SNQ2* | 201 | chrIV:465175-465685 | 257.9 | 198.4 | 212 | 235.3 | 250.2 |
| 31456 | Pronounced peak | 659 | chrX: 607186-607520 | 161.3 | 132.4 | 459 | 155.4 | 128.4 |
| 32533 | Hyper-ChIPable | 25 | chrI:142832-143070 | 63.8 | 27 | 645 | 51.0 | 25.7 |
| 33822 | Negative peak | 1314 | chrMito:13785-23783 | 382.3 | 228.9 | 30 | 111.0 | 8.7 |

^a^ Consolidated peak Region 201 was upstream of gene *SNQ2*, known for higher expression induced by Yrr1 in response to 4NQO (Cui *et al*. 1998; Le Crom *et al*. 2002; Onda *et al*. 2004), therefore peak instances within Region 201 are likely to represent true binding sites of Yrr1 for transcription activation of *SNQ2*. Region 201 showed pronounced peaks with high values for all the five-shape metrics (Table S4; an example is shown in Table S5 and Figure S2 A, B, C). Similarly, peak instances within Region 659 also showed high values for peak shape metrics (Table S4; an example is shown in Table S5 and Figure S2 D, E, F), therefore may represent true binding sites as well. Region 659 was upstream of gene *RPL43B*, not previously known to be regulated by Yrr1 or involved in 4NQO responses, therefore may be a novel finding based on peak shape metrics similar to Region 201. In addition, all the tested parameter sets of CisGenome and MACS2 identified peaks within Region 201 and 659 for most of the six conditions (combination of *YRR1* allele and growth medium) (Table S3). This further supports that peak instances within these two regions are likely to represent true binding sites. On the other hand, two regions that are less likely to represent true binding sites were also shown to contrast. Peak instances in Region 25 (e.g. Figure S3 A, B, C) overlap with a highly expressed gene *EFB1*, which encodes translation elongation factor 1 beta and showed FPKM 1076-1803 across all the conditions examined in RNA-Seq in this study. Therefore, peak instances within Region 25 are probably false positives due to hyper-ChIPability (Teytelman *et al*. 2013). Negative peak instances (regions enriched in input compared to ChIP) were identified within Region 1314 (e.g. Figure S3 D, E, F), and the shape metrics of an example negative peak instance calculated by flipping ChIP and input was shown. Some shape metrics of peak instances in Regions 25 and 1314 showed considerably lower values (highlighted by underlining) than those in Regions 201 and 659. Based on the assumption that lower values of the five shape metrics are linked to false positive peak instances, two sets of thresholds (Table S6) were used to select peak instances more likely to represent true binding sites because they show more pronounced shapes like those in Region 201 and 659.

Table S6

**Table S6**. Sets of thresholds used to select peaks with high confidence of being true binding sites of Yrr1. Peak instances must show greater values than the thresholds for all the five shape metrics to pass the set of thresholds.

| **Set of thresholds (minima)** | **Summit height** | **Summit difference** | **Summit distance (bp)** | **Rise from background mean** | **Rise from 2^nd^ summit** |
| --- | --- | --- | --- | --- | --- |
| Set 1 | 120 | 80 | 150 | 100 | 80 |
| Set 2 | 100 | 60 | 120 | 80 | 60 |

Table S7

**Table S7**. Consolidated peak regions with peak instances passing threshold set 1 and 2, as well as the genetic features overlapping with corresponding narrow peak regions.

| **Consolidated peak region** | |  | **Number of instances passing threshold set** | |  | **Overlapping feature** | |
| --- | --- | --- | --- | --- | --- | --- | --- |
| **ID** | **Coordinates** |  | **Set 1** | **Set 2** |  | **Name** | **FPKM range^a^** |
| 201 | chrIV:454940-465905 |  | 33 | 34 |  | *YDR010C* | 0-60 |
| 659 | chrX:606408-607949 |  | 28 | 32 |  | None | - |
| 527 | chrVII:893849-894948 |  | 5 | 11 |  | *SNG1, YPP1* | 30-49, 31-57 |
| 305 | chrIX:248423-249499 |  | 1 | 3 |  | *tS(UGA)I* | Likely high |
| 118 | chrIII:1-1187 |  | 11 | 13 |  | *TEL03L* | Telomere |
| 728 | chrXII:10953-11969 |  | 6 | 11 |  | *TEL12L* | Telomere |
| 1005 | chrXIV:783435-784407 |  | 4 | 6 |  | *TEL14R* | Telomere |
| 414 | chrVI:4432-5365 |  | 4 | 4 |  | *TEL05L* | Telomere |
| 595 | chrVIII:556144-557249 |  | 0 | 4 |  | *TEL08R* | Telomere |
| 727 | chrXI:666053-666899 |  | 0 | 2 |  | *TEL11R* | Telomere |
| 772 | chrXII:488797-489549 |  | 0 | 2 |  | *RDN5-6* | Likely high |
| 70 | chrII:300064-301549 |  | 0 | 1 |  | *RPL4A* | 1248-1913 |
| 357 | chrV:191894-192768 |  | 0 | 1 |  | *SPC25* | 44-61 |
| 494 | chrVII:609707-610256 |  | 0 | 1 |  | None | - |
| 526 | chrVII:882500-883857 |  | 0 | 1 |  | *TDH3* | 26692-35198 |
| 669 | chrX:702018-702894 |  | 0 | 1 |  | *RPS4A* | 1224-1789 |
| 716 | chrXI:519459-520723 |  | 0 | 1 |  | *UTH1* | 2486-3313 |
| 754 | chrXII:341016-341941 |  | 0 | 1 |  | *MIM2* | 140-375 |
| 770 | chrXII:485176-486011 |  | 0 | 1 |  | *RDN5-5* | Likely high |
| 1007 | chrXV:28429-29364 |  | 0 | 1 |  | *HPF1* | 30-104 |
| 1033 | chrXV:274082-275272 |  | 0 | 1 |  | *tS(GCU)O* | Likely high |
| 1064 | chrXV:642006-642721 |  | 0 | 1 |  | None | - |
| 1116 | chrXVI:296884-297456 |  | 0 | 1 |  | None | - |

^a^ FPKM range across all the combination of alleles and media in RNA-Seq. FPKM values are not available for rRNA (*RDN5-5*) and tRNA genes (*tS(UGA)I*, *tS(GCU)O*) since reads matched to those genes were excluded from RNA-Seq analysis. However, those genes are likely to have high FPKM had their reads not been excluded, due to the high cellular abundance of those RNAs. FPKM values were not calculated for telomeres because they are not transcribed.

Table S8

**File S8 Rong_2016_SI_TableS8_YPglyc_rna.xlsx**

**Table S8**. Differential expression data from RNA-Seq, analyzed using DESeq2 for all the tested loci and comparisons among alleles and growth media YPD and YPglycerol. In each comparison ‘condition_A/condition_B’, condition_A is the denominator and condition_B is the numerator when calculating fold change. Values of log2(fold) change for genes that were significant as determined by the z-score.

Table S9

**File S9 Rong_2016_SI_TableS9_all_GO_terms.xlsx**

**Table S9** GO term enrichment across yeast with different alleles of Yrr1 in 4NQO and glycerol compared to YPD.

# Supporting Figures mentioned in supporting text

**
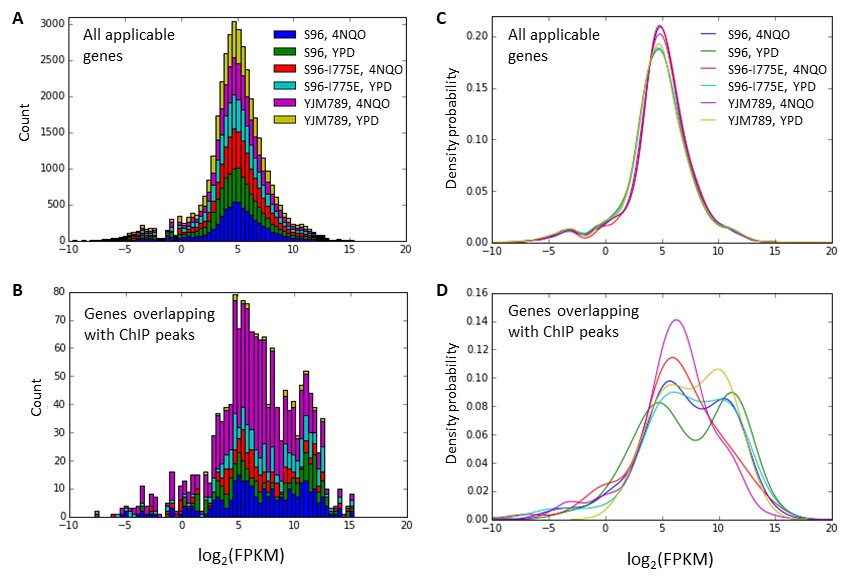
Figure S1**. Loci that overlap with narrow peak regions (defined in Materials and Methods and Supporting Information) of Yrr1 ChIP tend to show higher expression levels. For plotting as log_2_-transformed values, zero-FPKM values were adjusted to random numbers between 0 and 0.1 (or -3.32 as log_2_ value) sampled from normal distribution, since minimum non-0 FPKM value in the whole RNA-Seq dataset was 0.1. Only loci showing non-0 FPKM before adjustment under at least one condition (combination of *YRR1* allele and growth medium) were plotted in attempt to exclude rRNA, tRNA and dubious open reading frames. **A**, **B**: histograms stacked by conditions showing counts of loci. **C**, **D**: fitted curves for histograms via Gaussian Kernel Density Estimation showing approximate fraction of loci for each condition. **A**, **C**: all the 6620 applicable loci. **B**, **D**: 933 loci that overlap with 888 narrow peak regions in Yrr1 ChIP identified by CisGenome and/or MACS2 (out of 1136 narrow peak regions in total). The color codes are the same between **A** and **C** and between **B** and **D**, respectively.

**
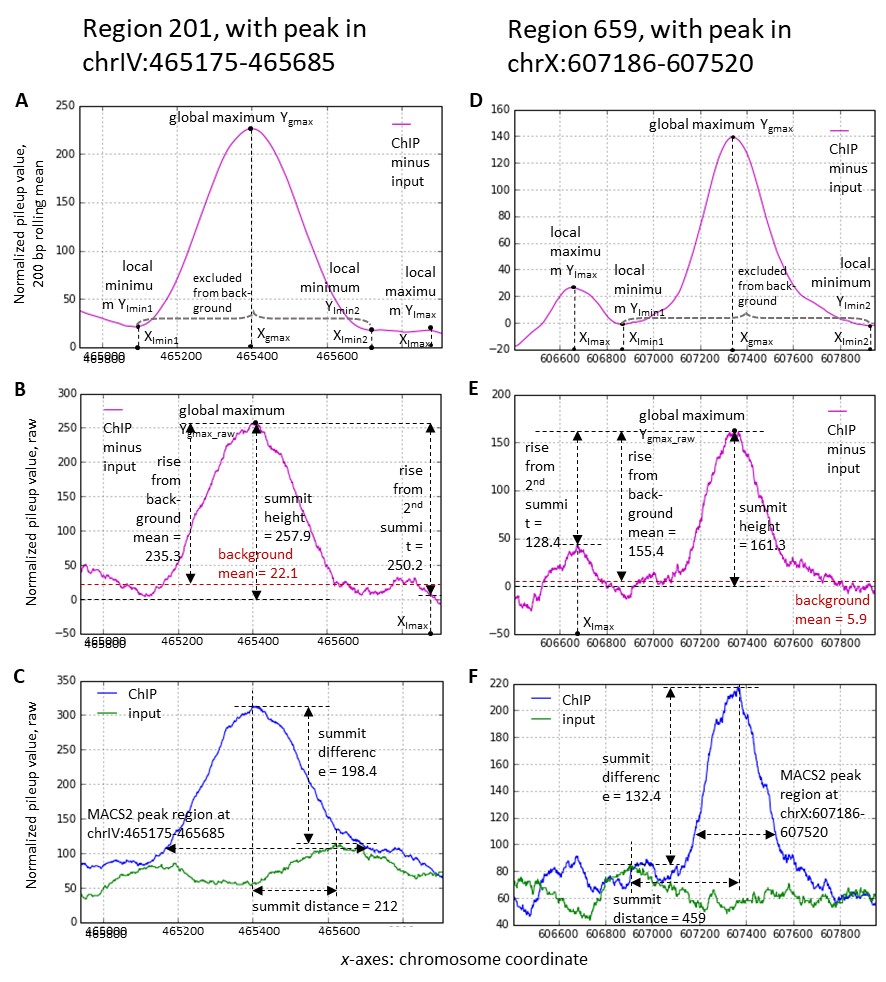
Figure S2**. Illustration of peak shape metrics on peak instances within consolidated peak regions 201 and 659, also as examples of peak instances likely to represent functional binding sites of Yrr1. Plotted are bedGraph pileup data generated by MACS2 using option --nomodel from ChIP replicate 3 and input of Yrr1^S96-I775E^ in 4NQO (mn_T6_ChIP3). **A**, **B**, **C**, region 201. **D**, **E**, **F**, region 659. **A**, **D**, rolling means per 200-bp window of normalized pileup values of ChIP minus input. **B**, **E**, normalized pileup values of ChIP minus input. **C**, **F**, normalized pileup values of ChIP and input.

**
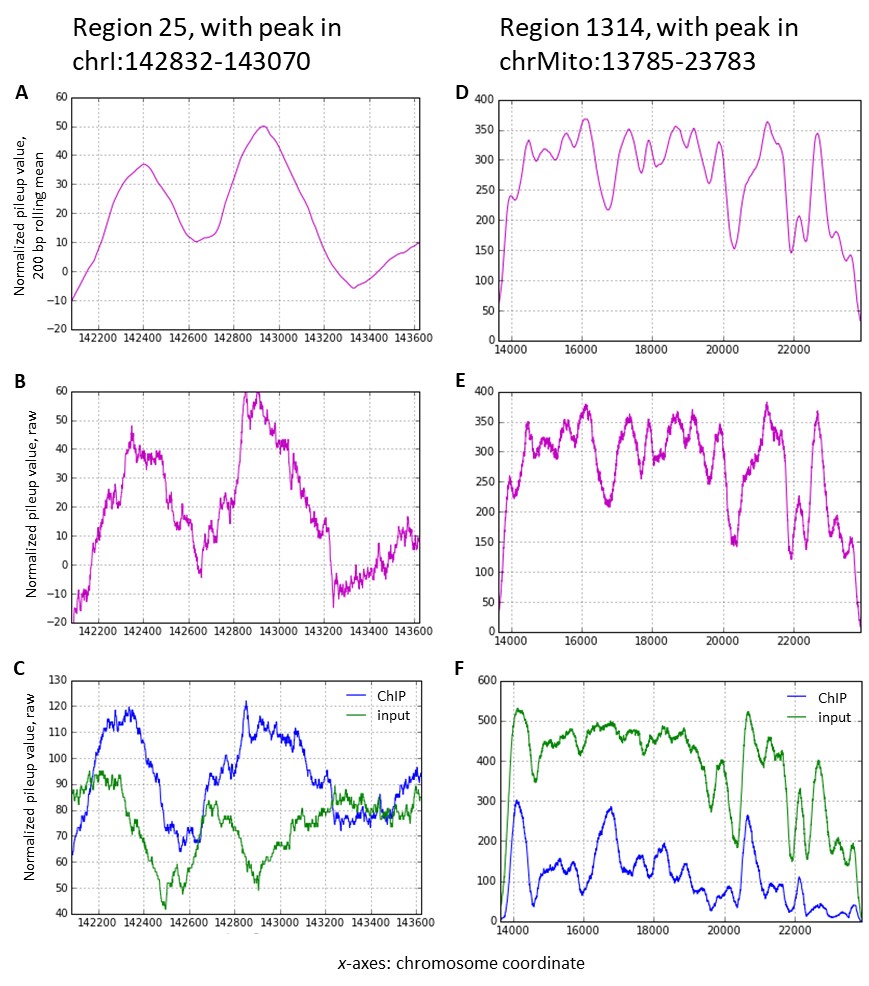
Figure S3**. Examples of peak instances unlikely to represent functional binding sites of Yrr1, within consolidated peak regions 25 and 1314. Plotted are bedGraph pileup data generated by MACS2 using option --nomodel from ChIP replicate 2 and input of Yrr1^S96-I775E^ in YPD (mn_T5_ChIP2). **A**, **B**, **C**, region 25. **D**, **E**, **F**, region 1314. **A**, **D**, rolling means per 200-bp window of normalized pileup values of ChIP minus input. **B**, **E**, normalized pileup values of ChIP minus input. **C**, **F**, normalized pileup values of ChIP and input.

**
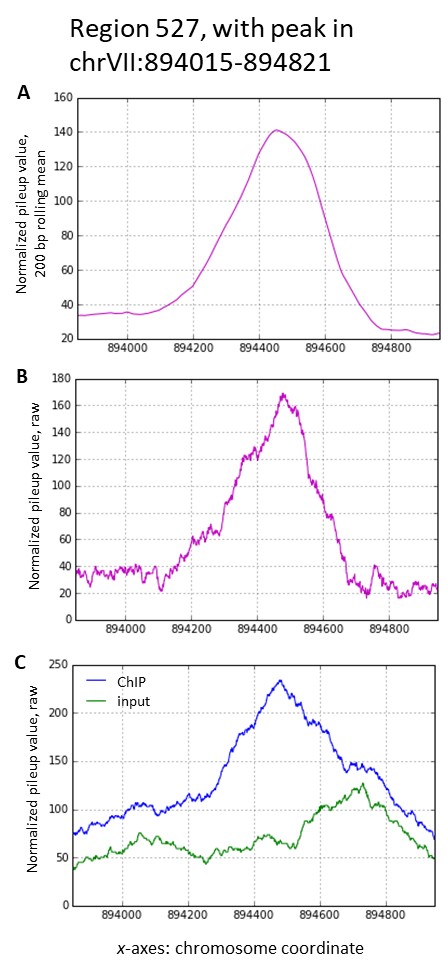
**

**Figure S4.** An example of the peak instances within consolidate peak region 527, identified as likely to represent functional binding sites of Yrr1 based on peak shape metrics. Plotted are bedGraph pileup data generated by MACS2 using option --nomodel from ChIP replicate 2 and input of Yrr1^YJM789^ in 4NQO (mn_T2_ChIP2). **A**, rolling means per 200-bp window of normalized pileup values of ChIP minus input. **B**, normalized pileup values of ChIP minus input. **C**, normalized pileup values of ChIP and input.

**
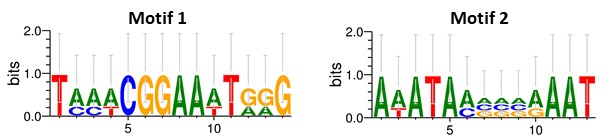
**

**Figure S5.** Two DNA motifs from the three high-confidence regions (Table 2) representing potential binding sites of Yrr1. The height of each nucleotide letter represents the posterior mean relative entropy and the error bars represent Bayesian 95% confidence intervals.

**
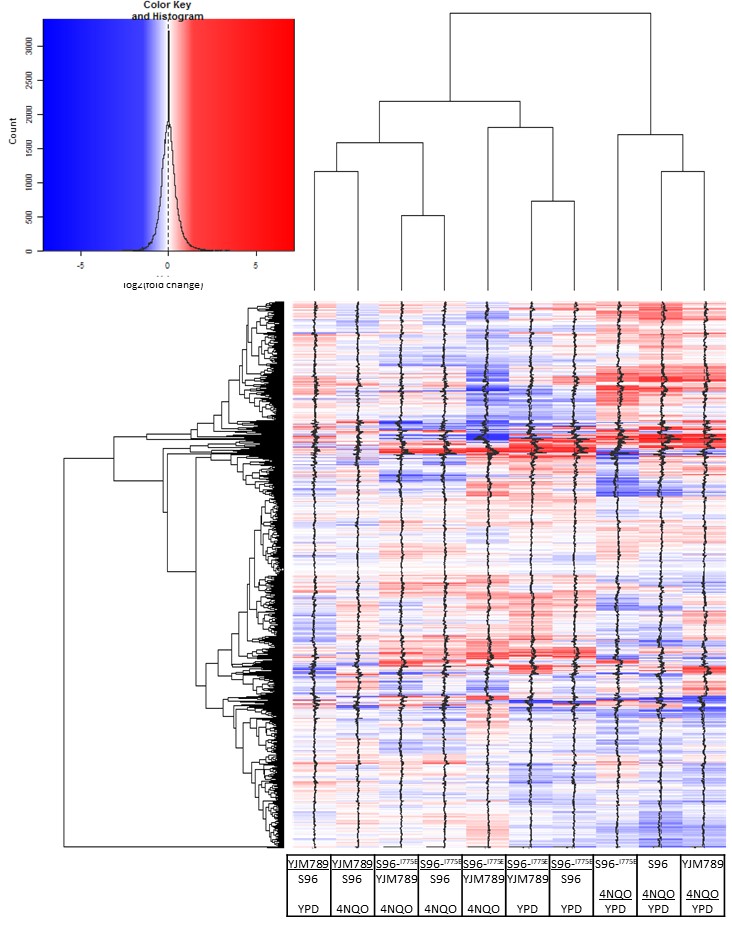
Figure S6**. Heatmap showing differential expression of all the tested loci and comparisons as outputs by DESeq2.

**
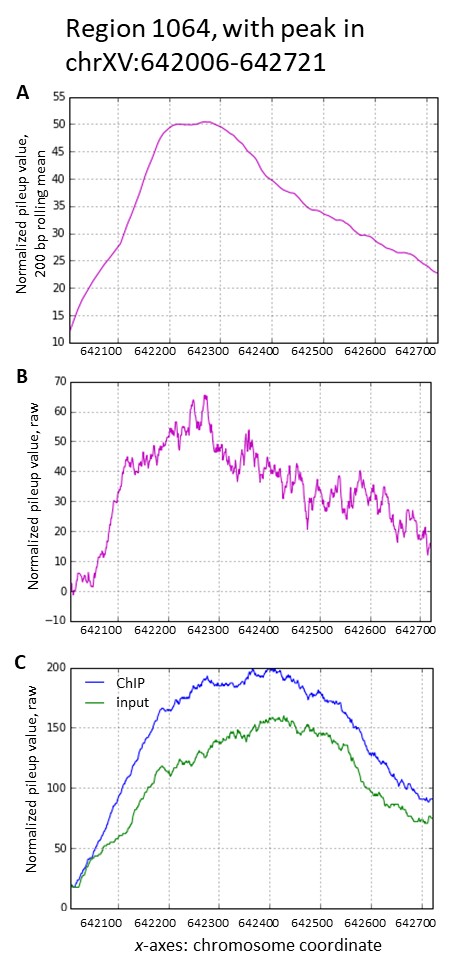
**

**Figure S7**. An example of the peak instances with notable input enrichment over nearby regions, within consolidate peak region 1064, upstream of *YRR1*. Plotted are bedGraph pileup data generated by MACS2 using option --nomodel from ChIP replicate 1 and input of Yrr1^S96^ in YPD (mn_T3_ChIP1). **A**, rolling means per 200-bp window of normalized pileup values of ChIP minus input. **B**, normalized pileup values of ChIP minus input. **C**, normalized pileup values of ChIP and input.

**
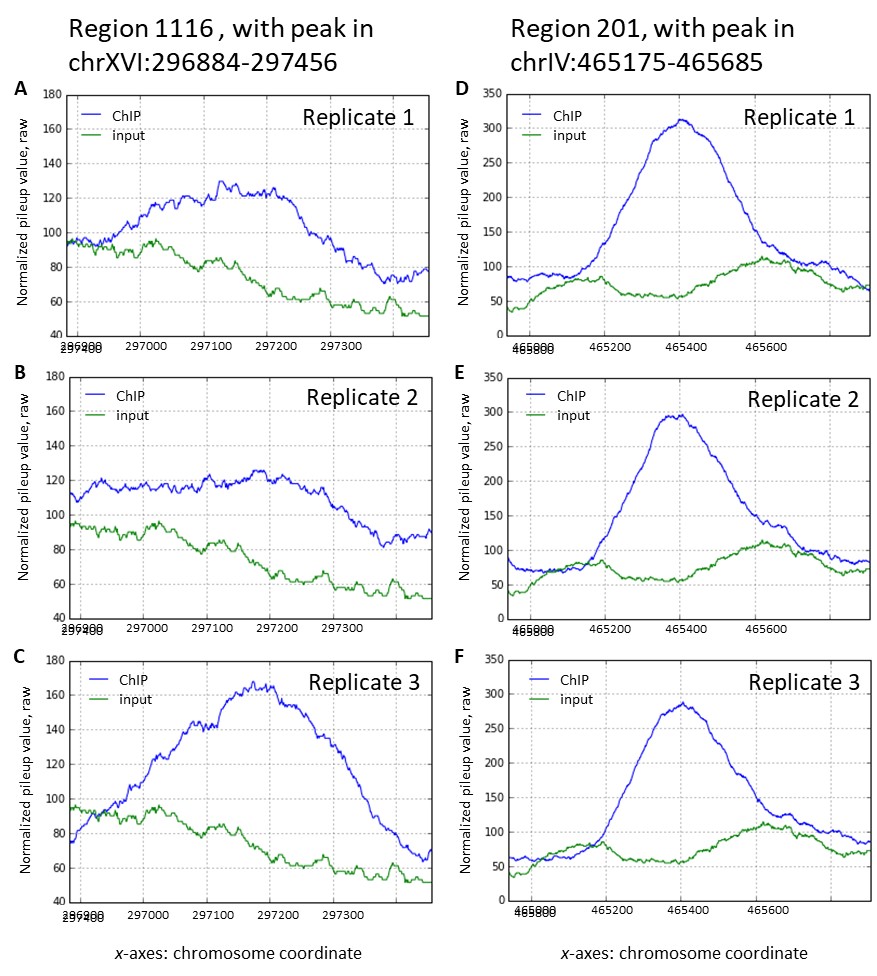
Figure S8**. Examples of different levels of variation in ChIP enrichment over input among replicates. Plotted are bedGraph normalized pileup data generated by MACS2 using option --nomodel for ChIP and input of Yrr1^S96-I775E^ in 4NQO (mn_T6). **A**, **B**, **C**, region 1116 as an example of high variation (inconsistency) among replicates. **D**, **E**, **F**, region 201 as an example of low variation (consistency) among replicates. **A**, **D**, ChIP replicates 1. **B**, **E**, ChIP replicates 2. **C**, **F**, ChIP replicates 3.

**
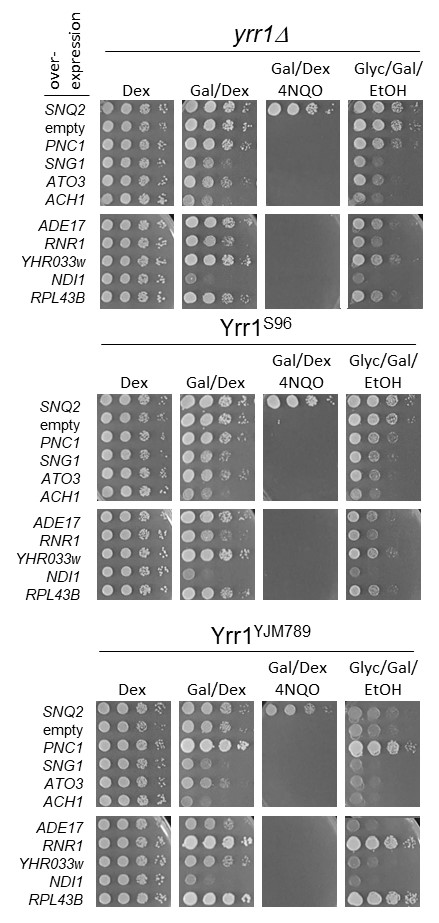
**

**Figure S9**. Growth assays of yeast overexpressing various gene. S288c (FY3 *yrr1Δ*) will different alleles of Yrr1 were transformed with either an empty plasmid (*yrr1*), or plasmids over-expressing genes under the control of the GAL promoter*.* Ten-fold serial dilutions of yeast grown in selective media to maintain both plasmids were spotted selective media for overexpression plasmids (pMORF) and plasmid driven Yrr1. Plates were incubated for three days and photographed.

**
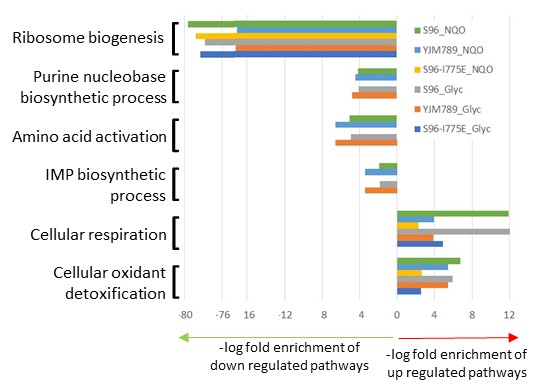
**

**Figure S10**. GO term enrichment comparison across different alleles of Yrr1 in YPD, 4NQO, Glyc. The -log of the p-value are graphed. GO:0098869 cellular oxidant detoxification, GO:0045333 cellular respiration, GO:0006188 IMP biosynthetic process, GO:0043038 amino acid activation, GO:0009113 purine nucleobase biosynthetic process, GO:0042254 ribosome biogenesis.

# End
